# The Dendritic Cell Response to Classic, Emerging, and Homeostatic Danger Signals. Implications for Autoimmunity

**DOI:** 10.3389/fimmu.2013.00138

**Published:** 2013-06-10

**Authors:** Paul M. Gallo, Stefania Gallucci

**Affiliations:** ^1^Laboratory of Dendritic Cell Biology, Department of Microbiology and Immunology, Temple Autoimmunity Center, Temple University School of Medicine, Philadelphia, PA, USA

**Keywords:** dendritic cells, autoimmunity, DAMPs, mitochondria, nanomaterial, acidosis, hypoxia, osmolarity

## Abstract

Dendritic cells (DCs) initiate and control immune responses, participate in the maintenance of immunological tolerance and are pivotal players in the pathogenesis of autoimmunity. In patients with autoimmune disease and in experimental animal models of autoimmunity, DCs show abnormalities in both numbers and activation state, expressing immunogenic levels of costimulatory molecules and pro-inflammatory cytokines. Exogenous and endogenous danger signals activate DCs to stimulate the immune response. Classic endogenous danger signals are released, activated, or secreted by host cells and tissues experiencing stress, damage, and non-physiologic cell death; and are therefore referred to as damage-associated molecular patterns (DAMPs). Some DAMPs are released from cells, where they are normally sequestered, during necrosis (e.g., heat shock proteins, uric acid, ATP, HMGB1, mitochondria-derived molecules). Others are actively secreted, like Type I Interferons. Here we discuss important DAMPs in the context of autoimmunity. For some, there is a clear pathogenic link (e.g., nucleic acids and lupus). For others, there is less evidence. Additionally, we explore emerging danger signals. These include inorganic materials and man-made technologies (e.g., nanomaterials) developed as novel therapeutic approaches. Some nanomaterials can activate DCs and may trigger unintended inflammatory responses. Finally, we will review “homeostatic danger signals,” danger signals that do not derive directly from pathogens or dying cells but are associated with perturbations of tissue/cell homeostasis and may signal pathological stress. These signals, like acidosis, hypoxia, and changes in osmolarity, also play a role in inflammation and autoimmunity.

## Introduction

Dendritic cells (DCs) are specialized antigen-presenting cells (APCs) that help maintain peripheral tolerance and orchestrate the adaptive immune response by presenting both endogenous and exogenous antigens and producing immune modulatory factors, such as costimulatory/inhibitory molecules and cytokines (Banchereau et al., 2000; Hammer and Ma, 2013). DCs reside in virtually all the tissues of our body in a predominantly antigen-capturing state and maintain immunologic tolerance by routinely migrating to the draining lymph nodes and presenting self-antigens to lymphocytes in a tolerogenic manner (Steinman et al., 2003; Reis e Sousa, 2006). Upon stimulation, DCs undergo a process of maturation/activation, which allows them to present in an immunogenic fashion the antigens captured in the periphery and initiate immune responses (Cella et al., 1997; Blander and Medzhitov, 2004). Danger signals are molecules released during infections (Janeway, 1989; Medzhitov and Janeway, 1997b) and/or tissue damage and cellular stress (Matzinger, 1994, 1998, 2002; Gallucci and Matzinger, 2001; Land and Messmer, 2012). Danger signals activate DCs and stimulate both the innate and adaptive immune response (**Box 1**).

Box 1 **Terms and definitions.**

In order to help our readers, here we define the terms we use throughout this review:

**Pathogen-associated molecular pattern:** molecular structures common to bacteria, viruses, or other microorganism, like LPS, flagellin, and peptidoglycan, that are able to activate DCs. These molecules are recognized by pattern recognition receptors (PRRs) expressed on both immune and non-immune cells. Since PAMPs are also expressed by the normal flora of mammalian mucosas, they are also called Microbial Associate Molecular Patterns (MAMPs).

**Damage-associated molecular pattern:** endogenous molecular structures that are normally contained within the cell interior and hidden from the immune system, and are liberated upon tissue damage. Examples include ATP, HSPs, and HMGB1. These molecules are recognized by a number of receptors, including PRRs, and are capable of inducing inflammation and immune responses in the absence of infection.

**Danger signal:** a danger signal may be any substance or event that is able to activate DCs and therefore initiate immune responses. For the purposes of this review, we have split the danger signals into three categories shown below. They all have in common an association with cellular stress and the ability to activate DCs.

**Classic danger signals:** we use the term “classic” to refer to danger signals that are not emerging or homeostatic (defined below). The classic danger signals are molecular species either associated with pathogens (PAMPs) or directly derived from tissue injury damage-associated molecular patterns (DAMPs) as described above, or secreted by activated immune cells as amplifiers of the immune activation.
**Emerging danger signals:** we use the term “emerging” to turn attention to new fields of research and technology that may pose new challenges to the immune system. Specifically, emerging danger signals include inorganic materials and man-made technologies (e.g., nanomaterials) often used as novel therapeutic strategies. These materials have the potential to activate DCs directly or indirectly by inducing tissue damage and release of DAMPs.
**Homeostatic danger signals:** with the term “homeostatic” we aim to highlight a less appreciated category of endogenous danger signals. Homeostatic danger signals generally represent perturbations in tissue steady state often as a result of inflammation. These perturbations include (but are not limited to) hypoxia, changes in acidity, or osmolarity, and metabolic stress.

**Figure 1:**
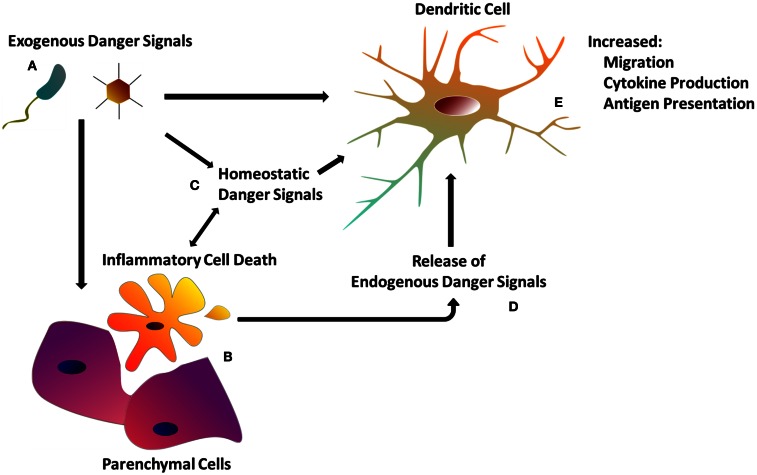
**The dendritic cell response to danger**. **(A)** Exogenous danger signals include pathogen-associated molecular patterns, as well as exogenous particulate matter. Exogenous danger signals activate DCs directly via pattern recognition receptors, or indirectly though tissue damage **(B)** and homeostatic perturbations **(C)**. **(B)** Inflammatory cell death as a result of tissue injury or programed necrosis causes the release of endogenous danger signals **(D)** (see Table 1) which also activate DCs (see Table 1). **(C)** Homeostatic perturbations such as those found in inflamed tissue (e.g., decreased pH, hypoxia) may also act as endogenous danger signals, influencing DC immune functions. **(E)** DCs integrate both exogenous and endogenous danger signals in order to orchestrate the appropriate immune response.

The recognition of tissue and cell damage – which may involve several types of innate immune cells beside DCs and cascades such as the complement – has been proposed to initiate multiple processes (Bianchi, 2007), including: (a) the development of inflammation, (b) the induction of innate effector functions leading to pathogen clearance, (c) the stimulation of adaptive immunity, which generates immunological memory for future encounters with the same antigen, and (d) the stimulation of tissue repair processes, necessary to reconstitute tissue integrity compromised by the infection or, in some cases, by the immune response itself (Stoecklein et al., 2012) (Figure 1).

Dendritic cells collaborate with other cells of the innate immune system to initiate what is often called “sterile inflammation and immunity;” that is, the activation of the innate and adaptive immune response in the absence of pathogens (Rock and Kono, 2008). Mechanisms of sterile inflammation are key to our understanding of clinical situations such as transplant rejection, tumor immunity, chronic inflammatory diseases, trauma-induced systemic inflammatory response syndrome (SIRS), and other inflammatory conditions like ischemia-reperfusion injury and atherosclerosis – in which immune responses develop when no overt infection is present. Autoimmune diseases, in which the adaptive immune system mounts an immune response against self-antigens, are also an important class of pathologies in which the role of infectious triggers for immunity is still controversial (Mills, 2011; Stranger and De Jager, 2012). The aim of this review is to analyze the danger signals that modulate DC functions and explore how those danger signals may contribute to the pathogenesis of autoimmunity.

## Dendritic Cells in Autoimmunity

The DC lineage comprises a complex population of several subsets – with recent advances demonstrating intricacies of gene transcription (Miller et al., 2012) and tissue localization (Gerner et al., 2012) that define each population. Indeed, DC subsets vary greatly in terms of lineage markers, cytokine production, and ability to stimulate different immune responses, largely depending on the tissue in which they reside or emigrate from [i.e., skin (Ginhoux et al., 2012) or gut (Rescigno, 2010)]. For simplicity, DCs are categorized as conventional DCs, strong APCs, or plasmacytoid DCs, the major producers of Type I Interferons (IFNs). DCs are either resident within the secondary lymphoid organs like lymph nodes and spleen, or migratory, coming into the lymph nodes from the peripheral tissues (Hammer and Ma, 2013).

The importance of DCs in the pathogenesis of autoimmune diseases is supported by the effects of their manipulation in experimental animal models. Indeed, the constitutive genetic depletion of DCs induces spontaneous development of autoimmunity characterized by autoantibodies against nuclear and tissue-specific antigens, multi-organ lymphocyte infiltration, and severe tissue damage, especially in the intestine (Ohnmacht et al., 2009). Therefore, DCs may be necessary for the establishment and maintenance of immunological self-tolerance (Reis e Sousa, 2006). Paradoxically, DCs also seem to be powerful inducers of autoimmunity. Mice in which DCs accumulate as a result of a DC specific defect in apoptosis also develop chronic lymphocyte activation and lupus-like systemic autoimmunity (Chen et al., 2006). Consequently, uncontrolled DC functions are sufficient to prime for autoimmune reactions. In many experimental models of autoimmunity, DCs accumulate in the secondary lymphoid organs and in the tissues targeted by the autoimmune process (Rosmalen et al., 2000; Kalled et al., 2001; Adachi et al., 2002; Colonna et al., 2006), although the causative mechanism for this accumulation remains unclear. Further support comes from studies demonstrating that the uptake of autoantibody-opsonized apoptotic cells increases DC presentation of self Ags in an inflammatory context (Frisoni et al., 2005). Moreover, *in vivo* priming with immunogenic (highly activated), self-antigen-loaded DCs induces, or accelerates autoimmunity (Bondanza et al., 2003; Eriksson et al., 2003), while the administration of tolerogenic DCs reduces disease and has been proposed as a potential therapeutic strategy in many models of autoimmune disease, such as type I diabetes (Feili-Hariri et al., 2002) and experimental autoimmune encephalomyelitis (EAE) (Menges et al., 2002; Toscano et al., 2010).

Dendritic cells may play a pathogenic role in autoimmunity by presenting self-antigens to T cells in an immunogenic fashion and by collaborating in the activation of autoreactive B cells. To do so, DCs have to be activated and express immunogenic costimulatory molecules and pro-inflammatory cytokines. Indeed, much evidence shows abnormally activated DC phenotypes in patients with different autoimmune diseases, as well as in murine models of autoimmunity (reviewed in Amodio and Gregori, 2012). In some autoimmune strains of mice, DCs show abnormalities even when generated in culture, far removed from the autoimmune microenvironment (i.e., DCs from young mice, before the onset of the disease) (Sriram et al., 2012). These results suggest a genetic defect intrinsic to DCs, leading to their excessive activation, possibly through an uncontrolled production of danger signals (Elkon and Stone, 2011). In other cases, abnormalities were present only in DCs *in vivo* or isolated *ex vivo* from diseased mice, therefore pointing to a direct association with the autoimmune process (Colonna et al., 2006; Melli et al., 2009) and suggesting that in some situations DC abnormalities are a consequence rather than the cause of the autoimmune environment. In either case, determining the role of danger signals in the activation of DCs is key to better understanding the pathogenesis of autoimmune disease. Knowledge of danger signals will also facilitate the identification of novel therapeutic approaches aimed at the prevention of autoreactive lymphocyte activation. For example, neutralization of stimuli that induce abnormal DC activation may be a far more physiologic strategy than relying on general suppression of the lymphocyte response, as is common with most current therapeutic protocols.

## The Extended Family of Danger Signals

The term “danger signal” was originally proposed by Polly Matzinger to indicate endogenous molecules released by stressed or necrotic cells, which are able to activate DCs (Matzinger, 1994, 1998; Gallucci et al., 1999; Gallucci and Matzinger, 2001). Five years earlier, Janeway (1989) had theorized that the innate immune system becomes activated by conserved molecular species expressed by evolutionarily distant microorganisms. These features were called pathogen-associated molecular patterns (PAMPs) and were proposed to trigger PRRs present on host cells (Medzhitov and Janeway, 1997a,b). With his innovative theory, Janeway (1992) updated the classic self-non-self-discrimination model of immunity by theorizing that the immune system can distinguish between self and infectious non-self, i.e., the pathogen (Medzhitov et al., 1997; Poltorak et al., 1998). As our understanding of the biochemical basis of the PRRs (e.g., TLRs, NLRs, RIG-I) has improved, it has become clear that host factors, derived from damaged tissues and cells, signal through the same receptors, serving as “danger signals” to stimulate immunity and therefore allow the immune system to discriminate what is dangerous or damaged from what is not, as previously theorized by Matzinger (1994, 2002) (Figure 1). These endogenous danger signals are a subset of what Seong and Matzinger (2004) named “DAMPs,” analogous to the nomenclature of Janeway. Presently the term “danger signal” has a broad meaning and includes very different families of molecules that activate DCs: either exogenous molecules, such as the PAMPs; or endogenous molecules released, activated, or secreted by host cells and tissues undergoing stress, damage, and non-physiological cell death, namely DAMPs (Matzinger, 1998, 2002) (Figure 1; Table 1). We support this inclusive nomenclature as it conveys the idea that both pathogens and trauma/stress are inducers of tissue and cell damage, which results in a pathologic status that is required for the activation of the innate immune system.

**Table 1:** **Endogenous danger signals**.

Signal	Source	Receptor/sensor(s)
Nucleic Acids	Dead/dying cells	TLR-7, -8, -9
Retroviral DNA	Cytoplasmic accumulation of endogenous retroviral DNA	STING
ATP, ADP, adenosine	Cell interior Dead/dying cells	P2X, P2Y A1, A2A, A2B, A3
	Mitochondria	
	Activated platelets	
Uric Acid	Nucleic acid breakdown	TLRs
	Dead/dying cells	CD14
		Inflammasome
HSPs	Cell interior	TLRs
	Dead/dying cells	CD91
	Mitochondria	
HMGB1	Cell Nucleus	TLRs
	Necrotic cells	RAGE
	Secreted by activated innate immune cells	CD24-Siglec10-TIM-3
		CXCR4
Type I IFNs	Virally infected cells	IFN receptors
	pDCs and mDCs	
Degradation products of the ECM	Extracellular matrix	TLR4 CD44 (hyaluronan)
MtDNA	Dead/dying cells	TLR9
	Mitochondria	
*N*-formyl peptides	Dead/dying cells Mitochondria	High affinity formal peptide receptors
Acidity	Perturbation in homeostasis Inflammation	Acid-sensing ion channels
	Secreted by tumor cells	Acid-sensing G-proteins
Osmolarity	Perturbation in homeostasis	mTOR
Hypoxia	Perturbation in homeostasis	Mitochondria

In the next section, we will review the DAMPs that activate DCs in autoimmunity, and we will then focus on danger signals that are less in the spotlight for immunologists, although already hypothesized in the original version of the Danger Model (Matzinger, 1994): the emerging and the homeostatic danger signals. Emerging danger signals are molecules that are newly developed by modern technology and that can activate DCs either by inducing cellular stress/damage or by attracting and carrying endogenous danger signals. Finally, we will review “homeostatic danger signals,” danger signals that do not derive directly from pathogens or dying cells but rather are associated with perturbations of tissue/cell homeostasis and may be considered signs of stress and non-physiological conditions. We will not review PAMPs since they have been subjects of many excellent reviews.

## Endogenous Danger Signals

Classic endogenous danger signals are molecules that activate DCs (Table 1; Figure 1; **Box 2**). As DCs are central to antigen presentation and the initiation of the immune response, it was first shown that DCs respond to endogenous danger signals released from dying cells (Gallucci et al., 1999; Sauter et al., 2000), explaining the adjuvant effects of dying cells when co-injected with antigen *in vivo* (Gallucci et al., 1999; Shi et al., 2000, 2003).

Box 2 **Primary vs. secondary endogenous danger signals.**

Endogenous danger signals can be divided in two categories depending on the trigger and on the cellular source.

**Primary endogenous danger signals:** endogenous molecules that are normally contained within the cell interior or present in an inactive form, hidden from the immune system, and mostly performing non-immune functions. They are released upon tissue damage and are able to activate DCs by triggering a number of receptors, including PRRs. They are the first initiators of sterile immunity. Examples are listed in Table 1.

**Secondary endogenous danger signals:** endogenous molecules that are actively secreted by immune cells upon activation to mediate innate and adaptive immune activation in an autocrine and paracrine manner. They fuel and direct both sterile and non-sterile immunity. Secondary endogenous danger signals include lymphocyte-derived activators of DCs such as CD40-L (Ridge et al., 1998) and granulysin (Zitvogel and Kroemer, 2010); neutrophil-derived alarmins (Yang et al., 2009) and pro-inflammatory cytokines such as TNF-α, Type I IFNs, and HMGB1.

Endogenous danger signals can be classified as “primary” when secreted by stressed cells or released by dying cells, or as “secondary” when produced by activated immune cells (Gallucci and Matzinger, 2001) (**Box 2**). Some cytokines act as both primary and secondary danger signals, when they are secreted by damaged/dying cells (**Box 2**). Many primary endogenous danger signals are released from the interior of the cell when the cell loses plasma membrane integrity upon necrosis (Rock and Kono, 2008; Sims et al., 2010). In contrast, the process of apoptosis keeps these pro-inflammatory signals contained and can actively up-regulate anti-inflammatory mediators (Behrens et al., 2007).

We will next describe some of the most studied DAMPs in autoimmunity. Covering all possible endogenous danger signals is not the purpose of this review: we will discuss a few signals in detail with specific reference to their role in autoimmune diseases (Table 1).

### Nucleic acids

Nucleic acids play a complex role in the pathogenesis of the autoimmune disease systemic lupus erythematosus (SLE). Nucleic acids are both the primary self-antigens and potent DAMPs, activating DCs through TLR7 and TLR9 and inducing the production of Type I IFNs (Barrat et al., 2005; Marshak-Rothstein and Rifkin, 2007). Nucleic acids often activate DCs either as part of an immune complex with autoantibodies (Leadbetter et al., 2002) or in complex with chaperones like heat shock proteins (HSP) and HMGB1. Oligonucleotides that inhibit the response to TLR7 and TLR9 ameliorate disease in lupus-prone mice (Barrat et al., 2007). Similarly the genetic deficiency of TLR7, or TLR7 and TLR9 combined prevent disease, while the single deficiency of TLR9 renders the disease worse, suggesting a double-edge effect of TLR9 (Santiago-Raber et al., 2009). In our hands, DCs from lupus-prone mice express constitutively high levels of Type I IFNs and IFN responsive genes and this IFN Signature was diminished by treatment with oligonucleotides inhibitory for TLR7 and TLR9, supporting a role for the response of DCs to nucleic acids in the excessive production of Type I IFNs in lupus (Sriram et al., 2012). The importance of TLR7 is further highlighted by the discovery that the Yaa translocation of a piece of chromosome X, containing 16 genes including TLR7, to the Y chromosome induces in male mice a severe lupus-like disease by creating a duplication of TLR7 (Pisitkun et al., 2006). Necrotic cells are a source of immunogenic nucleic acids (Eloranta et al., 2009) as well as neutrophils undergoing NETosis (Garcia-Romo et al., 2011).

### Endogenous viral elements

A subset of PAMPs that can also be considered a form of special endogenous danger signals are endogenous viral elements (EVEs). EVEs are sections of viral DNA that have integrated into the mammalian genome during evolution (Stoye, 2012). The best characterized EVEs come from retroviruses, but may also derive from other viruses (Katzourakis and Gifford, 2010). Recent evidence demonstrates that defects in controlling retroviral elements are linked to autoimmunity. Indeed, mice deficient in the cytoplasmic three prime repair exonuclease 1 (Trex1) (formerly DNase III) express elevated IFN alpha (IFNα) levels in many organs and develop inflammatory myocarditis in the absence of viral infection (Morita et al., 2004). *Trex1*-deficient (*Trex1*−/−) murine embryonic fibroblasts accumulate endogenous retroviral DNA in the cytosol which induces excessive Type I IFN production via STING (Yang et al., 2007; Stetson et al., 2008; Gall et al., 2012). Loss-of-function mutations in *Trex1* are present in patients with Aicardi–Goutières syndrome as well as other autoimmune diseases such as familial chilblain lupus (Lee-Kirsch et al., 2007a; Gunther et al., 2009) and SLE (Lee-Kirsch et al., 2007b; Hur et al., 2008). This evidence suggests that cytoplasmic DNA derived from endogenous retroviruses is an endogenous danger signal that, if not scavenged by Trex1, can trigger an intracellular nucleic acid-sensing machinery (Pichlmair and Reis e Sousa, 2007; Thompson et al., 2011), leading to excessive production of the pro-autoimmunogenic Type I IFNs (Elkon and Stone, 2011). Although DCs are activated by Type I IFNs (Gallucci et al., 1999), and might therefore become hyperactivated, and thus initiate the autoimmune process, phenotypic, or specific functional abnormalities of DCs in Trex1−/− mice or in patients with Aicardi–Goutières syndrome have not yet been reported.

### ATP

Other forms of nucleic acids acting as danger signals are the intracellular single nucleotides ATP and UTP. Extracellular ATP concentration is typically kept very low (10 nM) by ectonucleotidases (Robson et al., 2006). Under pathologic conditions, however, local concentrations of ATP can be increased – when liberated from damaged tissue or when released by an active process such as platelet or phagocyte activation (Zeiser et al., 2011). These adenosine derivatives activate DCs (Marriott et al., 1999) by stimulating the high affinity purinergic receptors P2Z/P2X_7_ (Mutini et al., 1999). ATP and UTP activate the inflammasome in collaboration with TLRs (Mariathasan et al., 2004) and mediate at least some of the sterile inflammation induced by necrotic cells (Iyer et al., 2009; Aymeric et al., 2012). P2X_7_-activated DCs have been shown to be crucial for the induction of tumor immunity (Ghiringhelli et al., 2009), the lack of P2X_7_ signaling decreases the severity of EAE (Sharp et al., 2008), and oxidized ATP (oxATP), an inhibitor of the ATP receptor P2rx7, ameliorated autoimmune type I diabetes and autoimmune encephalitis in mice (Lang et al., 2010). Apoptotic cells can also release small amounts of ATP through the pannexin family of plasma membrane channels. At this low concentration, ATP binds low affinity purinergic receptors, including P2Y_2_ and P2X_7_ on phagocytes and recruits them to accelerate non-inflammatory clearance of apoptotic cells (Elliott et al., 2009). In a model of allergic lung inflammation, P2Y_2_ is required for myeloid DC and eosinophil migration, although this receptor does not seem to be involved in DC maturation (Muller et al., 2010). Therefore, the ATP molecule may represent an important DAMP, conserved from prokaryotes (Atarashi et al., 2008) to plants (Heil et al., 2012) to mammals (Aymeric et al., 2012), acting as an activation signal at high doses when released by damaged cells, while at lower doses it may act as a “find-me” signal that helps housekeeping clearance of apoptotic cells and prevents immune activation (Elliott et al., 2009).

### Uric acid

Uric acid was one of the first DAMPs to be discovered and is partly responsible for the adjuvant properties of dying cells (Shi et al., 2003). Uric acid is a product of purine metabolism and is normally soluble inside cells. When released in the extracellular space uric acid precipitates and forms insoluble crystals of monosodium urate (MSU) capable of activating DCs *in vitro* and of acting as an adjuvant *in vivo* (Shi et al., 2003; Rock et al., 2010). Extracellular uric acid is highly inflammatory when it accumulates in joints to cause gout (Rock et al., 2013) or when it is released in large amounts during anti-cancer therapy causing tumor lysis syndrome (Muslimani et al., 2011). The inflammatory nature of uric acid, as well as of other crystals like silica and alum, is in part due to their ability to activate the inflammasome (Martinon et al., 2006) with the extracellular delivery of endogenous ATP, the stimulation of IL-1β, and other pro-inflammatory cytokines, and the induction of reactive oxygen species (ROS) (Schorn et al., 2010; Riteau et al., 2012). These crystals may activate the inflammasome by causing direct cell damage to phagocytes, including DCs, inducing phagolysosome rupture (Hornung et al., 2008). Indeed, membrane fragments of these organelles can activate several responses, including polyubiquitination, autophagy, and pyroptosis and may play a role in host defense against intracellular parasites (Hilbi, 2009). Excessive activation of the inflammasome has been strongly linked to a group of hereditary autoinflammatory diseases called cryopyrin associated periodic syndromes (Lamkanfi and Dixit, 2012) but it may also play a role in more classic autoimmune diseases such as multiple sclerosis (MS) as mice deficient for components of the inflammasome (Nlrp3 and ASC) are protected from the development of EAE (Gris et al., 2010; Shaw et al., 2010).

### Heat shock proteins

Heat shock proteins are the most abundant proteins in cells, responsible for maintaining the correct folding of nascent and misfolded proteins, and of proteins that must remain inactive for long periods. HSPs are up-regulated in response to tissue stressors such as elevated temperature, osmotic shock, and cytotoxic agents (Srivastava, 2002). During cellular necrosis, HSPs are released into the extracellular space where they activate the innate immune response by triggering TLRs (Vabulas et al., 2002). As HSPs are protein chaperones, they also actively participate in DC antigen processing and presentation to initiate adaptive immunity (Srivastava, 2002). Srivastava’s group showed that necrotic cells, but not apoptotic cells, release many HSPs such as HSP70, HSP90, calreticulin, and GP96 (Basu et al., 2000), which may activate immunity through the common receptor CD91 (Binder et al., 2000). In contrast, other HSPs, like the human heat shock protein 60, induce DC maturation and promote a Th1 phenotype via TLR4 (Flohe’ et al., 2003). Like HMGB1 and autoantibodies, HSPs may contribute to the development of autoimmunity not only as endogenous adjuvants but also as chaperones that physically deliver autoantigens to APCs (Biswas et al., 2006). This is the mechanism by which HSP70 promotes autoimmunity in a mouse model of diabetes (Liu et al., 2003; Millar et al., 2003). Furthermore, autoantibodies against HSPs are found in SLE patients and allelic variants of the *Hsp70* genes are significantly associated with SLE (Furnrohr et al., 2010) and with several other autoimmune diseases such as MS (Ramachandran and Bell, 1995), Crohn’s disease (Debler et al., 2003), Grave’s disease (Ratanachaiyavong et al., 1991), and insulin-dependent diabetes mellitus (Pociot et al., 1993).

### High mobility group box protein 1

High mobility group box protein 1 (HMGB1) is a well-established endogenous danger signal (Lotze and Tracey, 2005), released by necrotic cells that have lost membrane integrity (Scaffidi et al., 2002) as well as secreted by phagocytic cells as a late mediator of inflammation (Gardella et al., 2002). HMGB1 normally has a nuclear localization and was originally described as a DNA binding protein (Javaherian et al., 1978; Bianchi et al., 1989), but a variety of other cellular functions have been attributed to HMGB1 largely depending on different post-translational modifications such as oxidation and hyperacetylation (Harris et al., 2012). Intracellular HMGB1 regulates gene transcription and autophagy (Tang et al., 2012). Extracellular HMGB1 acts as a classic DAMP when released by necrotic cells, or as a secondary danger signal when secreted by macrophages and DCs in response to LPS and TNF-α (Yang et al., 2005; Bianchi, 2007). Autocrine HMGB1 mediates DC activation and the ability to induce Th1 polarization (Dumitriu et al., 2005). The secretion of HMGB1 by monocytic cells is mediated by hyperacetylation of HMGB1, which localizes it to the cytoplasm where, upon a secondary signal, it is liberated into the extracellular compartment (Bonaldi et al., 2003).

HMGB1 is significantly increased in the serum of patients with brain and myocardial ischemia (Goldstein et al., 2006) and in septic patients in which it mediates the late phase of septic shock (Wang et al., 1999). HMGB1 is recognized by many receptors, like the receptor for advanced glycation end products (RAGE) (Kokkola et al., 2005) as well as by TLR2 and TLR4 (Park et al., 2004), CD24-Siglec10, and the recently reported TIM-3 (Chiba et al., 2012). When bound to the chemokine CXCL12, HMGB1 acts as a chemokine to recruit leukocytes to the site of inflammation by binding CXCR4 (Schiraldi et al., 2012). The shift from functioning as a chemokine to acting as a danger signal depends on its redox state: intracellular HMGB1 has three cysteines that are in a reduced state (all-thiol state); when it is secreted or released, the all-thiol HMGB1 can bind to CXCL12 and chemoattract unless two cysteines are oxidized, forming a disulfide bond and promoting HMGB1 danger signal functions (Yang et al., 2013). During apoptosis HMGB1 is kept intracellular, and uptake of apoptotic cells induces release of oxidized HMGB1 so that its pro-inflammatory activities are neutralized. Possible functions of this fully oxidized state are still under investigation (Yang et al., 2013).

Evidence suggests that HMGB1 participates in the pathogenesis of many autoimmune diseases like Rheumatoid Arthritis (RA), SLE, EAE, and diabetes (Zhang et al., 2009; Harris et al., 2012). For example, RA patients have increased levels of HMGB1 in the serum and synovial fluid that decreases upon therapy-induced amelioration of joint inflammation (Zetterstrom et al., 2008). Similarly, HMGB1 blockade ameliorates arthritis in animals, while its administration in the joints induces arthritis (Kokkola et al., 2003). In SLE patients, serum HMGB1 levels, as well as anti-HMGB1 autoantibody titers, positively correlate with disease activity (Jiang and Pisetsky, 2008; Abdulahad et al., 2011). HMGB1-containing nucleosomes from apoptotic cells have been shown to induce secretion of pro-inflammatory cytokines and expression of costimulatory molecules in macrophages and DCs and the administration of HMGB1-nucleosome complexes in mice induces lupus-like autoantibodies (Urbonaviciute et al., 2008; Urbonaviciute and Voll, 2011). In MS patients, extracellular HMGB1 is increased in the cerebrospinal fluid (CSF) and microglia and macrophages expressing cytosolic HMGB1 are increased in MS active lesions (Andersson et al., 2008). In mice, HMGB1 drives neuroinflammation in EAE and the inhibition of HMGB1 signaling with a neutralizing antibody ameliorates disease (Robinson et al., 2013). Clinical and experimental evidence also suggest a role for HMGB1 in the pathogenesis of type I diabetes. In diabetes-prone NOD mice, HMGB1 blockade significantly inhibited insulitis progression and delayed diabetes onset, decreased the number, and maturation of DCs in the pancreatic lymph nodes, and increased the number of regulatory T cells (Han et al., 2008). Once diabetes has developed, hyperglycemia can induce HMGB1 secretion (Yao and Brownlee, 2010; Dandona et al., 2013) and HMGB1 may contribute to the nephropathy and the vascular complications of diabetes by fueling inflammation and promoting tissue damage, especially in the kidney (Lin et al., 2012). Higher levels of serum HMGB1 were associated in type 1 diabetes patients with a higher risk of mortality, cardiovascular disease (Nin et al., 2012a), and kidney damage (Nin et al., 2012b).

In summary, HMGB1 is a powerful danger signal that plays a complex role in the pathogenesis of autoimmune diseases. Blockade of HMGB1 has thus far proven to be beneficial in ameliorating a number of types of experimental autoimmunity, making its therapeutic potential very promising. Further investigation is still needed to dissect the role of HMGB1 as an activator of DCs in terms of its multiple functions as a cytokine, chemokine, transcription regulator, and possible functions yet to be discovered.

### Type I interferons

Type I IFNs activate DCs *in vitro* and act as adjuvant *in vivo* (Gallucci et al., 1999). They are secreted by virally infected cells (Taniguchi and Takaoka, 2002) as a primary danger signal to alert both neighboring tissue cells and local immune cells to the presence of a viral infection. They are also secreted by immune cells as an amplifier of innate immunity (Liu, 2005). Type I IFNs (IFNs α and β) mediate the activation of DCs to promote adaptive immune responses such as cross-priming and isotype switching of responding murine B cells toward IgG2 (Theofilopoulos et al., 2005). Type I IFNs are particularly important in autoimmunity and especially in SLE in which an IFN Signature is present – an abnormally high expression of Type I IFN responsive genes in immune cells and tissues (Baechler et al., 2003; Bennett et al., 2003; Crow et al., 2003; Elkon and Stone, 2011). The hyper-activation of Type I IFNs may play a role in the early and most acute phases of disease because the IFN Signature is common in pediatric patients (Bennett et al., 2003) and in adults with central nervous system involvement and nephritis (Baechler et al., 2003). Supporting this notion, we have recently discovered that myeloid DCs and pDCs from lupus-prone Sle1,2,3 mice express an IFN Signature before the onset of autoimmunity. Even when generated in culture from bone marrow precursors, depleted of mature T and B cells, macrophages, and DCs, and thus, severed from the *in vivo* pro-autoimmune environment, DCs from Sle123 mice expressed an IFN Signature (Sriram et al., 2012). These results indicate that DCs are an independent cellular source of the pathogenic danger signals implicated in lupus (Elkon and Stone, 2011; Sriram et al., 2012). Since polymorphisms in genes that are part of the signaling pathway of Type I IFNs, such as IRF5, IRF7, and STAT4, are associated with a higher risk of developing SLE (Harley et al., 2009), the over-expression of Type I IFNs in these patients may be the result of the combination of genetic predisposition to produce high levels of Type I IFNs and exposure to danger signals that stimulate IFN production (Elkon and Stone, 2011; Niewold, 2011).

### Degradation products of the extracellular matrix

During tissue damage, not only are cells stressed and dying but components of the extracellular matrix (ECM) are disrupted leading to the production of low molecular weight degradation products such as hyaluronic acid (HA) and heparan sulfate, which have been shown to activate DCs and initiate inflammation (Termeer et al., 2002; Shirali and Goldstein, 2008; Brennan et al., 2012). Moreover, other components of the ECM such as fibrinogen, fibronectin extra-domain A (EDA), biglycan, and tenascin-C are up-regulated in response to tissue injury, and they all act as danger signals involved in the pathogenesis of RA (Goh and Midwood, 2011). Many of these molecules have been found increased in RA tissues; administration of danger signals, such as fibronectin EDA or tenascin-C, induces joint inflammation *in vivo*, while mice deficient in tenascin-C show a rapid resolution of inflammation (Goh and Midwood, 2011), indicating an important role for ECM molecules as initiator/amplifiers of the autoimmune process in RA.

## Emerging Exogenous Danger Signals

The classic exogenous danger signals originate from pathogens (PAMPs), but exogenous particles not derived from microbes, such as silica, crystals, and nanoparticles can also induce DC activation and inflammatory disease. Indeed, it has long been known that inhalation of fine particulate material can cause inflammatory and fibrotic lung disease (i.e., pneumoconiosis). The inflammatory nature of non-living particulate matter, like crystals and nanoparticles, may stem in part from their hydrophobic character (Seong and Matzinger, 2004). Moreover, nanoparticles are preferentially taken up by phagocytes (Dobrovolskaia et al., 2008), including DCs, in which they cause destabilization and rupture of the phagolysosome (Hornung et al., 2008), leading to inflammasome activation (Cassel et al., 2008). Therefore, extracellular inorganic material may directly damage DCs, leading to inflammasome activation and DC maturation. This is the proposed mechanism of action for alum, a widely used adjuvant in human vaccines (Eisenbarth et al., 2008). Because of their immunogenic potential, it is important to consider the emerging use of nanomaterials to target DCs for novel therapeutics and their possible autoimmunogenic side effects.

### Nanomaterials

The term nanoparticle may apply to almost any material with individual units at the nano scale. Nanomaterials can be generated from organic (e.g., liposomes, organic polymers) or inorganic materials (e.g., gold, silver) and engineered to display a molecule of interest or contain a therapeutic payload. Using nanoparticles and synthetic material systems, it may be possible to modulate the immune response through direct manipulation of DCs (Elamanchili et al., 2007).

The usefulness of pro-inflammatory nanoparticles has been demonstrated in a number of fields (Fadeel, 2012; Fadeel et al., 2012). For example, nanoparticle-based co-delivery of antigens and danger signals directly to DCs may be a promising strategy to direct anti-tumor immune responses (Kim and Mooney, 2011), as well as useful in next-generation vaccine adjuvants (Demento et al., 2009). However, because materials designed as next-generation therapies are sometimes capable of inducing DC-mediated inflammation similarly to PAMPs or DAMPs (Koike et al., 2008; Li et al., 2010), nanomaterials may raise concerns for safety (Dobrovolskaia and McNeil, 2007; Stern and McNeil, 2008). For example, nanoparticle debris from prosthetic alloys can activate the inflammasome (Caicedo et al., 2009), nanoparticles are known to modulate various forms of inflammatory cell death (Andón and Fadeel, 2013), and some nanomaterials are strong activators of complement (Salvador-Morales et al., 2006).

Additionally, it is now clear that nanoparticles do not stay “naked” when introduced *in vivo* – they become coated by biomolecules (e.g., proteins) to form what is termed a bio-corona (Fadeel, 2012). The composition and biophysical properties of the bio-corona is likely to have major implications for safety and effectiveness of future therapies (Aggarwal et al., 2009). The protein bio-corona first develops as a “soft” coat which eventually becomes a compact “hard” shell, becoming difficult to remove (Mohamed et al., 2012). Proteins within the hard coat are therefore aggregated and may become unfolded or modified (for example, citrullinated), possibly resulting in DCs recognizing altered self-antigen as danger signals, although evidence for this is still lacking (Fadeel, 2012).

The study of nanoparticle characteristics is also likely to uncover as yet unknown characteristics of danger signals. With the ability to control the characteristics and the delivery of the “danger signal,” we may better understand the immune response to those factors. For example, size of the nanoparticle seems to determine which DC populations interact with an injected particle: namely, larger particles require peripheral DCs for transport into the draining lymph nodes, whereas smaller particles act on lymph node resident cells directly (Manolova et al., 2008). Large particles also tend to be more inflammatory, with smaller particles often eliciting no reaction at all (Fadeel, 2012), possibly because larger sized particles more potently destabilize the phagolysosome.

Although most studies focus on the pro-inflammatory nature of particulate matter, some nanomaterials may suppress immune function (Mitchell et al., 2009; Zolnik et al., 2010) or can be engineered to evade the innate immune system (Moghimi, 2002). Indeed, gold has anti-inflammatory properties that have been exploited for many years in treatment for RA and gold nanoparticles are being engineered as novel treatments for a number of inflammatory diseases (Ulbrich and Lamprecht, 2010). Recently, Yeste et al. used gold nanoparticles to generate tolerogenic DCs in a murine model of EAE by delivering a tolerogenic compound in combination with oligodendrocyte antigen to DCs. These DCs in turn induced the generation of regulatory T cells which mitigated disease progression (Yeste et al., 2012).

Although gold may have some anti-inflammatory properties and may be useful as a delivery system, it also has many toxic side effects, earning itself a black box warning and precluding it as a practical therapy. Indeed, heavy metals can cause autoimmune-like syndromes (Schiraldi and Monestier, 2009) and therefore should be implemented cautiously as therapeutics. However, it is important to note that nanoparticles are being developed which are both biocompatible and biodegradable. For example, liposomes have been used as a delivery system to successfully treat experimental murine arthritis (Metselaar et al., 2003; Sethi et al., 2013), and biodegradable nanoparticles have been used to localize and target T cells (Fahmy et al., 2007). Nanoparticles coated with glycans have been used to target antigen to DCs (García-Vallejo et al., 2013), but the therapeutic use of DC-targeted nanoparticles in spontaneous autoimmunity remains largely unexplored (Ulbrich and Lamprecht, 2010). Nanomaterials therefore represent a promising strategy for next-generation therapy in the autoimmune diseases and an important area for future investigation to insure their safety and efficacy.

## Homeostatic Endogenous Danger Signals

**Figure 2:**
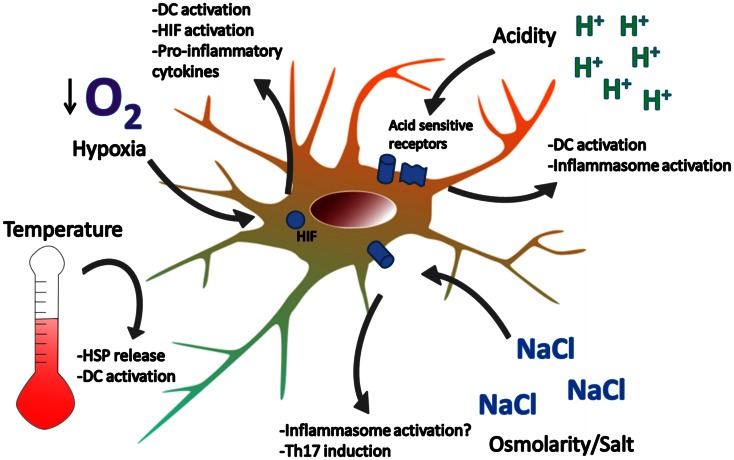
**Examples of homeostatic danger signals**. Localized perturbations in homeostasis are common in infection, ischemia-reperfusion injury, and inflammation associated with autoimmune disease. Extreme temperatures can cause DC activation and cell death. Heat induces the release of HSPs which can activate DC. Hypoxia is sensed by HIF and leads to DC activation, NF-kB signaling, and cytokine production. Acidity is sensed by acid-sensing ion channels and acid-sensing G-proteins, leading to DC activation. Hypotonicity activates the inflammasome in macrophages and high salt concentrations promote Th17 responses. How DCs respond to changes in osmolarity remains unclear.

So far, we have briefly described the DAMPs investigated under the classic paradigm – cell stress/death from tissue damage, possibly induced by trauma, infections or foreign materials, provides endogenous danger signals, which propagate the immune response (Figure 1). But a number of investigations suggest the existence of endogenous danger signals that do not derive from damaged or dying cells. Rather, they are signals that alert immune cells, and possibly non-immune cells, about perturbations in the steady state of the cellular microenvironment, and we refer to these as “homeostatic” danger signals (Figure 2). Indeed, the immune system is closely tied to metabolic homeostasis (reviewed in Odegaard and Chawla, 2013; Pearce and Pearce, 2013). These homeostatic danger signals may include conditions often characteristic of chronically inflamed tissues such as localized acidosis, changes in osmolarity, decreased oxygen tension (hypoxia), oxidative stress with ROS, and other metabolic disturbances – all possible causes of cellular stress as hypothesized in the original Danger Model (Matzinger, 1994). Homeostatic changes also result from bacterial growth and acute tissue damage, further supporting the notion that cells may sense homeostatic changes as danger. Some of these homeostatic perturbations directly activate DCs, because DCs have specific sensors for these perturbations, while others lead to expression of more classic DAMPs: an example of the latter is the up-regulation of HSP like HSP70 by DCs in response to hyperthermia (40 °C), resulting in up-regulation of costimulatory molecules, pro-inflammatory cytokines, and T cell stimulation (Knippertz et al., 2011).

### Acidity

Physiologic pH is normally held within relatively narrow range (pH 7.35–7.45). During localized inflammation (e.g., infection, arthritic joint) or tissue ischemia (e.g., lupus nephritis, MS lesions) extracellular pH can reach pH values as low as 5.5 (Treuhaft and McCarty, 1971; Nedergaard et al., 1991; Simmen and Blaser, 1993).

A significant amount of research on cell functions in acidic conditions comes from the cancer field. Tumor cells tend to use glycolysis for energy generation even when there is adequate oxygen supply – the Warburg effect (Cardone et al., 2005). The inefficiency of glycolytic metabolism, the active secretion of acid, and the poor perfusion in solid tumors is thought to contribute to an acidic microenvironment. Acidic microenvironments increase the invasiveness and metastatic potential of some cancers as a result of remodeling of the ECM (Martinez-Zaguilan et al., 1996; Rofstad et al., 2006) and activation of inflammatory processes that have been shown to promote tumor survival (Grivennikov, 2013).

Several studies have indicated that acidity might act as a danger signal. For example, extracellular acidification can promote activation of the inflammasome and secretion of IL-1β by monocytes (Jancic et al., 2011) and seems to activate neutrophils through the activation of PI3K-Akt and ERK pathways (Trevani et al., 1999; Martinez et al., 2006). Additionally, non-immune cells may also produce inflammatory cytokines in response to extracellular acidification (Shime et al., 2008; Ichimonji et al., 2010) or may alter cell death programs (Jouan-Lanhouet et al., 2012).

Acidity in the extracellular microenvironment was initially proposed to inhibit DC differentiation (Gottfried et al., 2006), but some work suggests that DCs are instead activated by acidosis (Martinez et al., 2006). Other evidence has shown that acidic environments might improve antigen uptake and antigen presentation in DCs, although the mechanisms are still unclear (Vermeulen et al., 2004). Recently, acid-induced activation of DCs was found to be in part due to acid-sensing ion channels (Tong et al., 2011), but our understanding of DC responses to protons remains limited (Lardner, 2001). Low pH may also be sensed by proton-sensing G protein-coupled receptors (Seuwen et al., 2006), as seen in responses from synovial cells (Christensen et al., 2005; Tomura et al., 2005). Acidity is a common feature of inflammation found in many autoimmune diseases (e.g. within the arthritic joint, nephritic kidney, ischemic lesions of MS). However, to our knowledge, it is still unclear whether pathways triggered by acidity are involved in DC immune functions in the context of autoimmune diseases.

### Osmolarity and Salt

Cell volume expansion, as a result of extracellular hypotonicity, may also act as a danger signal. In response to hypotonic osmotic challenge lymphocytes first swell, then undergo a process termed regulatory volume decrease (RVD) (Garber and Cahalan, 1997; Cahalan et al., 2001). A reciprocal process occurs in hypertonic environments called regulatory volume increase (RVI). RVD is largely the result of the activity of potassium and chloride channels, with efflux of KCl giving way to water efflux as a result of decreased intracellular osmolarity. In some cases KCl efflux also leads to activation of other ion channels and cellular depolarization, thus having major implications in fundamental cellular processes like metabolism and cell death. Indeed, a similar process is seen during apoptosis. Apoptotic cell volume decrease seems to be due to the activation of the same potassium and chloride channels (Maeno et al., 2000, 2006). Cellular responses resulting from homeostatic signals are complicated by the observation that ion channels form complexes with integrins and may signal together (Arcangeli and Becchetti, 2006).

Earlier studies demonstrated that hypotonicity influence immune cell function, for example in neutrophil shedding of L-selectin (Kaba and Knauf, 2001). Mammalian target of rapamycin (mTOR), in conjunction with mitochondrial metabolism, may also be an important sensor for osmotic stress, as well as other homeostatic signals (Desai et al., 2002; Kwak et al., 2012). DCs are able to pinocytose, uptake fluids from the extracellular compartment as a way to sample for antigens and danger signals. This process is very efficient in immature/resting DCs which can uptake fluids up to one thousand fold their volume (Sallusto et al., 1995). This large flow of fluids requires a tight control of the DC volume, and therefore DCs express aquaporins for this purpose (de Baey and Lanzavecchia, 2000). Inhibition of aquaporins leads to sudden increase in cell volume, which may ultimately result in DC cell death. The idea that hypotonicity may be regarded as a danger signal has recently gained credence with the finding that hypotonic solutions activate the NLRP3 inflammasome in macrophages via the efflux of K^+^ and Cl^−^ ions and RVD (Compan et al., 2012). The role of alterations of cell volume in DC physiopathology remains largely unclear.

In contrast to hypotonicity, high salt concentrations do not seem to activate inflammasome activation, as hypertonic solutions are not able to induce caspase-1-dependent release of cytokines IL-18 and IL-1β (Compan et al., 2012). However, high salt concentrations may promote development of other pro-inflammatory conditions such as those associated with T helper 17 cells (Th17). Osmotic shock due to high salt was known to induce the production of IL-8 from human peripheral blood mononuclear cells (Shapiro and Dinarello, 1995), but recently two groups have demonstrated that high salt concentrations may promote pathogenic Th17 responses (Kleinewietfeld et al., 2013; Wu et al., 2013) and a high salt diet exacerbates EAE (Wu et al., 2013). These studies suggest that elevated salt concentrations, similar to levels found in tissues of animals on high salt diets, may exacerbate autoimmune reactions. It is still unclear how high salt concentrations affect DCs *in vivo* in the context of autoimmunity.

### Hypoxia

Decreased oxygen tension is another characteristic disturbance of inflamed or damaged tissue (Nizet and Johnson, 2009). Innate immune cells are thought to be better than adaptive immune cells at surviving in such low oxygen conditions (Sica et al., 2011), and a number of signaling pathways are relatively well known (Gloire et al., 2006; Semenza, 2009). In normoxic conditions, the α subunits of hypoxia-inducible factor (HIF), HIF-1α, and HIF-2α, are constantly produced and promptly degraded. Under hypoxic conditions, HIF-α accumulates, forms a dimer with the constitutively expressed HIF-1β subunit within the nucleus, and regulates transcription via hypoxia-responsive elements (HREs). Important gene targets are involved in adaptive response to hypoxia and include glycolytic enzymes, erythropoietin, and vascular endothelial growth factor.

A number of lines of evidence suggest that hypoxia acts as a homeostatic danger signal. Low oxygen levels leads to the generation of ROS, affecting both metabolism and mediators of inflammation. Furthermore, it is well established that HIF-1α signaling is closely tied to NF-kB signaling. In neutrophils, survival in hypoxic microenvironments, such as those found in infected tissue, is mediated by HIF-1α-dependent NF-kB activation (Walmsley et al., 2005). In a number of cell types, including DCs, hypoxia-induced HIF-1α prompts expression of TLR2 and TLR6 (Kuhlicke et al., 2007). Additionally, macrophages with a conditional deficiency in HIF-1α are compromised in their ability to clear bacteria (Cramer et al., 2003; Peyssonnaux et al., 2005), and TLR ligands like LPS may induce the HIF-NF-kB axis even under normoxic conditions (Spirig et al., 2010). Indeed, hypoxia-independent HIF induction may occur via other mechanisms, including in response to bacterial siderophores (Hartmann et al., 2008). Finally, hypoxia is also known to modulate cytokine production via HIF expression, inducing production of pro-inflammatory cytokines (Jeong et al., 2003; Nizet and Johnson, 2009) and enhancing the production of IFN-β in response to viral infection (Hwang et al., 2006).

Studies of DC responses to hypoxia are at times conflicting. It is unclear if hypoxia in itself activates DCs (Jantsch et al., 2008; Wang et al., 2010), and hypoxia may enhance (Jantsch et al., 2008) or inhibit (Mancino et al., 2008) LPS-induced costimulatory molecule expression. However, hypoxia does seem to augment DC production of pro-inflammatory cytokines, particularly in response to LPS (Jantsch et al., 2008; Mancino et al., 2008; Blengio et al., 2013). Similar activation has been demonstrated in human DCs associated with the inflamed joints of patients with juvenile idiopathic arthritis (Bosco et al., 2011).

HIF-1α expression is associated with a number of inflammatory and autoimmune diseases, including within the synovium of RA patients (Hollander et al., 2001) and in the muscle from patients with dermatomyositis and systemic sclerosis (Konttinen et al., 2004). HIF-1α is important for the development of B cells, with HIF-1α deficiency in chimeric mice leading to autoimmunity (Kojima et al., 2002). To our knowledge, the role of HIF signaling in DCs in the context of autoimmunity remains relatively unexplored.

## Mitochondria

As a number of excellent reviews on mitochondria-derived danger signals have recently been published (Arnoult et al., 2011; Krysko et al., 2011; Galluzzi et al., 2012; Rath and Haller, 2012), we can only briefly touch on this important subject. Mitochondria are key in both the recognition and propagation of danger signals and we discuss them last because, as we describe in this section, mitochondria belong to more than one category of danger signal. As ancient symbionts, mitochondria harbor a number of molecules that have bacterial origin and therefore can be considered PAMPs. At the same time, these molecules are released from the cell interior upon cell stress/death and therefore they act as DAMPs and propagate the inflammatory response. Mitochondrial-derived danger signals include mitochondrial DNA (mtDNA), which contains CpG motifs capable of stimulating TLR9, *N*-formyl peptides which mimic bacterial peptides, and ATP as described above (Arnoult et al., 2011). The double nature of mitochondria as PAMPs and DAMPs brings the models of Janeway and Matzinger together (Seong and Matzinger, 2004), showing how the immune system co-evolved with his “enemy within” (Zhang et al., 2010). Moreover, as mitochondria are the cell power-house, they are key players in programs of cell death and in controlling homeostatic perturbations (Desai et al., 2002; Galluzzi et al., 2012).

Mitochondrial-derived danger signals are associated with the inflammatory response upon tissue damage and have been implicated in the inflammatory response to trauma. Severe traumatic injury may result in SIRS, a life threatening condition in which extensive tissue injury leads to systemic inflammation and shock reminiscent of sepsis (Stoecklein et al., 2012). Indeed, trauma patients who have sustained major tissue injury have elevated levels of circulating mtDNA which contributes to the resultant sterile shock (Zhang et al., 2010).

Mitochondrial-derived CpGs are also an important danger signal in autoimmune disease. For example, injecting mtDNA into the joints of mice induces myeloid cell-mediated arthritis, but injection of nuclear DNA does not (Collins et al., 2004). The authors of this study also found mtDNA in the synovial fluid of patients with RA, but not from health controls (Collins et al., 2004). Recently, a mitochondrial-derived protein related to HMGB1, called mitochondrial transcription factor A, has been found to synergize with CpG to induce the production of Type I IFNs by pDCs (Julian et al., 2012).

Furthermore, as mitochondria are central to metabolism and cellular energy production, they represent a key sensor of homeostatic perturbations (Desai et al., 2002) and are known to regulate multiple cell death programs – linking homeostatic signals to cell death and release of danger signals (Galluzzi et al., 2012). Importantly, mitochondrial control of cell death includes the execution of both apoptosis and regulated necrosis, the latter a form of inflammatory cell death now known to propagate the immune response to pathogens (Vandenabeele et al., 2010). Regulated necrosis leads to the liberation of many danger signals, including those we have outlined in this review.

Perturbations in homeostasis or mitochondrial function also lead to the generation of ROS, which are an important danger signals in their own right. ROS are normal byproducts of respiration, but may also function as signaling molecules and antimicrobial agents under controlled conditions (Valko et al., 2007); as well as a danger signals under pathologic conditions (Galluzzi et al., 2012). Cells defend themselves against toxic ROS buildup using a multitude of antioxidants and enzymes including superoxide dismutase and catalase (Dröge, 2002). Superoxide dismutase converts oxygen radicals to hydrogen peroxide and oxygen; and catalase converts hydrogen peroxide to water and oxygen. However, when oxidative stress overwhelms these antioxidant mechanisms the result is activation of cell death programs (Galluzzi et al., 2012). In the context of autoimmune diseases like SLE, increased ROS may contribute to pathogenesis via modification of self-antigens, thereby increasing their auto-reactivity, and by promoting cell death resulting in the release of DAMPs (Perl et al., 2004; Oates and Gilkeson, 2006).

In addition to being a source and sensor of danger signals, mitochondrial dysfunction may directly influence the course of autoimmune and autoinflammatory diseases. This seems to be due to the participation of mitochondria in regulating the inflammasome and cell death. Indeed, inflammasome activation in the context of monogenic autoinflammatory diseases has been suggested to cause mitochondrial dysfunction (Escames et al., 2011). Mitochondrial dysfunction and subsequent cell death, in response to oxidative stress, may have a predominant role in the propagation of MS (Witte et al., 2010). Mitochondria from T cells of SLE patients also seem to be deregulated – having an elevated baseline transmembrane potential, predisposing them to necrotic cell death (Gergely et al., 2002; Perl et al., 2012) and other metabolic disturbances (Wu et al., 2012). Continued investigation of mitochondria at the cross-roads between metabolism and inflammation will very likely be important in our understanding of autoimmunity.

## Conclusion and Future Perspectives

The immune response is often described as a double-edge sword. Too little immunity leads to uncontrolled infections, while too much immunity results in inflammatory damage, autoinflammatory disease, and some forms of autoimmunity. The delicate balance needed to keep immunity within this narrow range is the result of complex biochemical interactions. A threshold likely exists whereby tonic factors suppress noise in the system and other factors amplify or regulate immunologic signals in order to have a quick and efficient immune response (Germain, 2012). Danger signals may be both initiators of the immune response, as well as amplifiers of the immune response as a result of tissue injury and cell death. Indeed, inflammatory cell death seems to be required for proper pathogen clearance (Cho et al., 2009; Upton et al., 2010). Another layer of complexity comes in the fact that some molecules do not always act as danger signals, but rather serve other functions (e.g., HMGB1 acts as a chemokine, HSP as a chaperone, mitochondria as a power-house) and may even inhibit the immune response or promote tissue repair (Castiglioni et al., 2011; Stocki and Dickinson, 2012).

Danger signals are therefore diverse in form and function. Indeed, there are a number of conceptual frameworks in which danger signals are characterized, all of which have been extremely useful (Janeway, 1989; Matzinger, 1994). Here we have outlined, to the best of our ability, both the classic, emerging and homeostatic danger signals that influence DC biology. Although we describe some non-classic because they do not directly derive from pathogens or dead/dying cells, they are in fact well established. Man-made technologies (nanomaterials) are simply a novel form of particulate matter, which has been known for many years to induce inflammatory disease. Furthermore, the notion that homeostatic perturbations (e.g., osmotic stress, oxidative stress) act as danger signals is supported by a large body of literature (See “Mitochondria”). These data suggest that the immune system, set to recognize tissue/cell damage, can use sensors for basic physical and chemical perturbations of the tissue microenvironment to detect early causes of damage, even before the release of DAMPs. While much is now know about DC responses to exogenous and endogenous danger signals, many questions remain unresolved. Indeed, we have discussed signals as if they act independently, but we have little information on how signals interact with each other to control the immune response. Future studies will determine how the sum of these functions influences the development of autoimmunity.

## Conflict of Interest Statement

The authors declare that the research was conducted in the absence of any commercial or financial relationships that could be construed as a potential conflict of interest.

## References

[B1] Abdulahad D. A. Westra J. Bijzet J. Limburg P. C. Kallenberg C. G. Bijl M. (2011). High mobility group box 1 (HMGB1) and anti-HMGB1 antibodies and their relation to disease characteristics in systemic lupus erythematosus. Arthritis Res. Ther. 13, R71 10.1186/ar3332 21548924 PMC3218880

[B2] Adachi Y. Taketani S. Toki J. Ikebukuro K. Sugiura K. Oyaizu H. (2002). Marked increase in number of dendritic cells in autoimmune-prone (NZW x BXSB)F1 mice with age. Stem Cells 20, 61–72 10.1634/stemcells.20-1-61 11796923

[B3] Aggarwal P. Hall J. B. McLeland C. B. Dobrovolskaia M. A. Mcneil S. E. (2009). Nanoparticle interaction with plasma proteins as it relates to particle biodistribution, biocompatibility and therapeutic efficacy. Adv. Drug Deliv. Rev. 61, 428–437 10.1016/j.addr.2009.03.009 19376175 PMC3683962

[B4] Amodio G. Gregori S. (2012). Dendritic cells a double-edge sword in autoimmune responses. Front. Immunol. 3:233 10.3389/fimmu.2012.00233 22876246 PMC3410601

[B5] Andersson A. Covacu R. Sunnemark D. Danilov A. Dal Bianco A. Khademi M. (2008). Pivotal advance: HMGB1 expression in active lesions of human and experimental multiple sclerosis. J. Leukoc. Biol. 84, 1248–1255 10.1189/jlb.1207844 18644848

[B6] Andón F. T. Fadeel B. (2013). Programmed cell death: molecular mechanisms and implications for safety assessment of nanomaterials. Acc. Chem. Res. 46, 733–742 22720979 10.1021/ar300020b

[B7] Arcangeli A. Becchetti A. (2006). Complex functional interaction between integrin receptors and ion channels. Trends Cell Biol. 16, 631–639 10.1016/j.tcb.2006.10.003 17064899

[B8] Arnoult D. Soares F. Tattoli I. Girardin S. E. (2011). Mitochondria in innate immunity. EMBO Rep. 12, 901–910 10.1038/embor.2011.157 21799518 PMC3166463

[B9] Atarashi K. Nishimura J. Shima T. Umesaki Y. Yamamoto M. Onoue M. (2008). ATP drives lamina propria T(H)17 cell differentiation. Nature 455, 808–812 10.1038/nature07240 18716618

[B10] Aymeric L. Apetoh L. Ghiringhelli F. Tesniere A. Martins I. Kroemer G. (2012). Tumor cell death and ATP release prime dendritic cells and efficient anticancer immunity. Cancer Res. 70, 855–858 10.1158/0008-5472.CAN-09-3566 20086177

[B11] Baechler E. C. Batliwalla F. M. Karypis G. Gaffney P. M. Ortmann W. A. Espe K. J. (2003). Interferon-inducible gene expression signature in peripheral blood cells of patients with severe lupus. Proc. Natl. Acad. Sci. U.S.A. 100, 2610–2615 10.1073/pnas.0337679100 12604793 PMC151388

[B12] Banchereau J. Briere F. Caux C. Davoust J. Lebecque S. Liu Y. J. (2000). Immunobiology of dendritic cells. Annu. Rev. Immunol. 18, 767–811 10.1146/annurev.immunol.18.1.767 10837075

[B13] Barrat F. J. Meeker T. Chan J. H. Guiducci C. Coffman R. L. (2007). Treatment of lupus-prone mice with a dual inhibitor of TLR7 and TLR9 leads to reduction of autoantibody production and amelioration of disease symptoms. Eur. J. Immunol. 37, 3582–3586 10.1002/eji.200737815 18034431

[B14] Barrat F. J. Meeker T. Gregorio J. Chan J. H. Uematsu S. Akira S. (2005). Nucleic acids of mammalian origin can act as endogenous ligands for Toll-like receptors and may promote systemic lupus erythematosus. J. Exp. Med. 202, 1131–1139 10.1084/jem.20050914 16230478 PMC2213213

[B15] Basu S. Binder R. J. Suto R. Anderson K. M. Srivastava P. K. (2000). Necrotic but not apoptotic cell death releases heat shock proteins, which deliver a partial maturation signal to dendritic cells and activate the NF-kappa B pathway. Int. Immunol. 12, 1539–1546 10.1093/intimm/12.11.1539 11058573

[B16] Behrens E. M. Sriram U. Shivers D. K. Gallucci M. Ma Z. Finkel T. H. (2007). Complement receptor 3 ligation of dendritic cells suppresses their stimulatory capacity. J. Immunol. 178, 6268–6279 17475855 10.4049/jimmunol.178.10.6268

[B17] Bennett L. Palucka A. K. Arce E. Cantrell V. Borvak J. Banchereau J. (2003). Interferon and granulopoiesis signatures in systemic lupus erythematosus blood. J. Exp. Med. 197, 711–723 10.1084/jem.20021553 12642603 PMC2193846

[B18] Bianchi M. E. (2007). DAMPs, PAMPs and alarmins: all we need to know about danger. J. Leukoc. Biol. 81, 1–5 10.1189/jlb.0306164 17032697

[B19] Bianchi M. E. Beltrame M. Paonessa G. (1989). Specific recognition of cruciform DNA by nuclear protein HMG1. Science 243, 1056–1059 10.1126/science.2922595 2922595

[B20] Binder R. J. Han D. K. Srivastava P. K. (2000). CD91: a receptor for heat shock protein gp96. Nat. Immunol. 1, 151–155 10.1038/77835 11248808

[B21] Biswas C. Sriram U. Ciric B. Ostrovsky O. Gallucci S. Argon Y. (2006). The N-terminal fragment of GRP94 is sufficient for peptide presentation via professional antigen-presenting cells. Int. Immunol. 18, 1147–1157 10.1093/intimm/dxl049 16772370

[B22] Blander J. M. Medzhitov R. (2004). Regulation of phagosome maturation by signals from toll-like receptors. Science 304, 1014–1018 10.1126/science.1096158 15143282

[B23] Blengio F. Raggi F. Pierobon D. Cappello P. Eva A. Giovarelli M. (2013). The hypoxic environment reprograms the cytokine/chemokine expression profile of human mature dendritic cells. Immunobiology 218, 76–89 10.1016/j.imbio.2012.02.002 22465745

[B24] Bonaldi T. Talamo F. Scaffidi P. Ferrera D. Porto A. Bachi A. (2003). Monocytic cells hyperacetylate chromatin protein HMGB1 to redirect it towards secretion. EMBO J. 22, 5551–5560 10.1093/emboj/cdg516 14532127 PMC213771

[B25] Bondanza A. Zimmermann V. S. Dell’Antonio G. Dal Cin E. Capobianco A. Sabbadini M. G. (2003). Cutting edge: dissociation between autoimmune response and clinical disease after vaccination with dendritic cells. J. Immunol. 170, 24–27 12496378 10.4049/jimmunol.170.1.24

[B26] Bosco M. C. Pierobon D. Blengio F. Raggi F. Vanni C. Gattorno M. (2011). Hypoxia modulates the gene expression profile of immunoregulatory receptors in human mature dendritic cells: identification of TREM-1 as a novel hypoxic marker in vitro and in vivo. Blood 117, 2625–2639 10.1182/blood-2010-06-292136 21148811

[B27] Brennan T. V. Lin L. Huang X. Cardona D. M. Li Z. Dredge K. (2012). Heparan sulfate, an endogenous TLR4 agonist, promotes acute GVHD after allogeneic stem cell transplantation. Blood 120, 2899–2908 10.1182/blood-2011-07-368720 22760779 PMC3466971

[B28] Cahalan M. D. Wulff H. Chandy K. G. (2001). Molecular properties and physiological roles of ion channels in the immune system. J. Clin. Immunol. 21, 235–252 10.1023/A:1010958907271 11506193

[B29] Caicedo M. S. Desai R. Mcallister K. Reddy A. Jacobs J. J. Hallab N. J. (2009). Soluble and particulate Co-Cr-Mo alloy implant metals activate the inflammasome danger signaling pathway in human macrophages: a novel mechanism for implant debris reactivity. J. Orthop. Res. 27, 847–854 10.1002/jor.20826 19105226

[B30] Cardone R. A. Casavola V. Reshkin S. J. (2005). The role of disturbed pH dynamics and the Na+/H+ exchanger in metastasis. Nat. Rev. Cancer 5, 786–795 10.1038/nrc1713 16175178

[B31] Cassel S. L. Eisenbarth S. C. Iyer S. S. Sadler J. J. Colegio O. R. Tephly L. A. (2008). The Nalp3 inflammasome is essential for the development of silicosis. Proc. Natl. Acad. Sci. U.S.A. 105, 9035–9040 10.1073/pnas.0803933105 18577586 PMC2449360

[B32] Castiglioni A. Canti V. Rovere-Querini P. Manfredi A. A. (2011). High-mobility group box 1 (HMGB1) as a master regulator of innate immunity. Cell Tissue Res. 343, 189–199 10.1007/s00441-010-1033-1 20835834

[B33] Cella M. Engering A. Pinet V. Pieters J. Lanzavecchia A. (1997). Inflammatory stimuli induce accumulation of MHC class II complexes on dendritic cells. Nature 388, 782–787 10.1038/42030 9285591

[B34] Chen M. Wang Y. H. Wang Y. Huang L. Sandoval H. Liu Y. J. (2006). Dendritic cell apoptosis in the maintenance of immune tolerance. Science 311, 1160–1164 10.1126/science.1122545 16497935

[B35] Chiba S. Baghdadi M. Akiba H. Yoshiyama H. Kinoshita I. Dosaka-Akita H. (2012). Tumor-infiltrating DCs suppress nucleic acid-mediated innate immune responses through interactions between the receptor TIM-3 and the alarmin HMGB1. Nat. Immunol. 13, 832–842 10.1038/ni.2376 22842346 PMC3622453

[B36] Cho Y. S. Challa S. Moquin D. Genga R. Ray T. D. Guildford M. (2009). Phosphorylation-driven assembly of the RIP1-RIP3 complex regulates programmed necrosis and virus-induced inflammation. Cell 137, 1112–1123 10.1016/j.cell.2009.05.037 19524513 PMC2727676

[B37] Christensen B. N. Kochukov M. Mcnearney T. A. Taglialatela G. Westlund K. N. (2005). Proton-sensing G protein-coupled receptor mobilizes calcium in human synovial cells. Am. J. Physiol. Cell Physiol. 289, C601–C608 10.1152/ajpcell.00039.2005 15829562

[B38] Collins L. Hajizadeh S. Holme E. Jonsson I.-M. Tarkowski A. (2004). Endogenously oxidized mitochondrial DNA induces in vivo and in vitro inflammatory responses. J. leukoc. Biol. 75, 995–1000 10.1189/jlb.0703328 14982943

[B39] Colonna L. Dinnall J. A. Shivers D. K. Frisoni L. Caricchio R. Gallucci S. (2006). Abnormal costimulatory phenotype and function of dendritic cells before and after the onset of severe murine lupus. Arthritis Res. Ther. 8, R49 10.1186/ar1911 16507174 PMC1526610

[B40] Compan V. Baroja-Mazo A. López-Castejón G. Gomez A. I. Martínez C. M. Angosto D. (2012). Cell volume regulation modulates NLRP3 inflammasome activation. Immunity 37, 487–500 10.1016/j.immuni.2012.06.013 22981536

[B41] Cramer T. Yamanishi Y. Clausen B. R. E. Förster I. Pawlinski R. Mackman N. (2003). HIF-1alpha is essential for myeloid cell-mediated inflammation. Cell 112, 645–657 10.1016/S0092-8674(03)00154-5 12628185 PMC4480774

[B42] Crow M. K. Kirou K. A. Wohlgemuth J. (2003). Microarray analysis of interferon-regulated genes in SLE. Autoimmunity 36, 481–490 10.1080/08916930310001625952 14984025

[B43] Dandona P. Ghanim H. Green K. Sia C. Abuaysheh S. Kuhadiya N. (2013). Insulin infusion suppresses while glucose infusion induces Toll-like receptors and high-mobility group-B1 protein expression in mononuclear cells of type 1 diabetes patients. Am. J. Physiol. Endocrinol. Metab. 304, 8 10.1152/ajpendo.00566.2012 23403945

[B44] de Baey A. Lanzavecchia A. (2000). The role of aquaporins in dendritic cell macropinocytosis. J. Exp. Med. 191, 743–748 10.1084/jem.191.4.743 10684866 PMC2195835

[B45] Debler J. Schiemann U. Seybold U. Mussack T. Landauer N. Ladurner R. (2003). Heat-shock protein HSP70-2 genotypes in patients with Crohn’s disease: a more severe clinical course with intestinal complications in presence of the PstI-polymorphism. Eur. J. Med. Res. 8, 120–124 12730033

[B46] Demento S. L. Eisenbarth S. C. Foellmer H. G. Platt C. Caplan M. J. Mark Saltzman W. (2009). Inflammasome-activating nanoparticles as modular systems for optimizing vaccine efficacy. Vaccine 27, 3013–3021 10.1016/j.vaccine.2009.03.034 19428913 PMC2695996

[B47] Desai B. N. Myers B. R. Schreiber S. L. (2002). FKBP12-rapamycin-associated protein associates with mitochondria and senses osmotic stress via mitochondrial dysfunction. Proc. Natl. Acad. Sci. U.S.A. 99, 4319–4324 10.1073/pnas.261702698 11930000 PMC123646

[B48] Dobrovolskaia M. A. Aggarwal P. Hall J. B. Mcneil S. E. (2008). Preclinical studies to understand nanoparticle interaction with the immune system and its potential effects on nanoparticle biodistribution. Mol. Pharm. 5, 487–495 10.1021/mp800032f 18510338 PMC2613572

[B49] Dobrovolskaia M. A. McNeil S. E. (2007). Immunological properties of engineered nanomaterials. Nat. Nanotechnol. 2, 469–478 10.1038/nnano.2007.223 18654343

[B50] Dröge W. (2002). Free radicals in the physiological control of cell function. Physiol. Rev. 82, 47–95 10.1152/physrev.00018.2001 11773609

[B51] Dumitriu I. E. Baruah P. Valentinis B. Voll R. E. Herrmann M. Nawroth P. P. (2005). Release of high mobility group box 1 by dendritic cells controls T cell activation via the receptor for advanced glycation end products. J. Immunol. 174, 7506–7515 15944249 10.4049/jimmunol.174.12.7506

[B52] Eisenbarth S. C. Colegio O. R. O’Connor W. Sutterwala F. S. Flavell R. A. (2008). Crucial role for the Nalp3 inflammasome in the immunostimulatory properties of aluminium adjuvants. Nature 453, 1122–1126 10.1038/nature06939 18496530 PMC4804622

[B53] Elamanchili P. Lutsiak C. M. E. Hamdy S. Diwan M. Samuel J. (2007). “Pathogen-mimicking” nanoparticles for vaccine delivery to dendritic cells. J. Immunother. 30, 378–395 10.1097/CJI.0b013e31802cf3e3 17457213

[B54] Elkon K. B. Stone V. V. (2011). Type I interferon and systemic lupus erythematosus. J. Interferon Cytokine Res. 31, 803–812 10.1089/jir.2011.0045 21859344 PMC3216059

[B55] Elliott M. R. Chekeni F. B. Trampont P. C. Lazarowski E. R. Kadl A. Walk S. F. (2009). Nucleotides released by apoptotic cells act as a find-me signal to promote phagocytic clearance. Nature 461, 282–286 10.1038/nature08296 19741708 PMC2851546

[B56] Eloranta M. L. Lovgren T. Finke D. Mathsson L. Ronnelid J. Kastner B. (2009). Regulation of the interferon-alpha production induced by RNA-containing immune complexes in plasmacytoid dendritic cells. Arthritis Rheum. 60, 2418–2427 10.1002/art.24686 19644885

[B57] Eriksson U. Ricci R. Hunziker L. Kurrer M. O. Oudit G. Y. Watts T. H. (2003). Dendritic cell-induced autoimmune heart failure requires cooperation between adaptive and innate immunity. Nat. Med. 9, 1484–1490 10.1038/nm960 14625544

[B58] Escames G. López L. C. García J. A. García-Corzo L. Ortiz F. Acuña-Castroviejo D. (2011). Mitochondrial DNA and inflammatory diseases. Hum. Genet. 131, 161–173 10.1007/s00439-011-1057-y 21735170

[B59] Fadeel B. (2012). Clear and present danger? Engineered nanoparticles and the immune system. Swiss Med. Wkly. 142:w13609 10.4414/smw.2012.13609 22736064

[B60] Fadeel B. Pietroiusti A. Shvedova A. (2012). Adverse Effects of Engineered Nanomaterials: Exposure, Toxicology, and Impact on Human Health. Boston: Academic Press

[B61] Fahmy T. M. Fong P. M. Park J. Constable T. Saltzman W. M. (2007). Nanosystems for simultaneous imaging and drug delivery to T cells. AAPS J. 9, E171–180 10.1208/aapsj0902019 17614359 PMC2751406

[B62] Feili-Hariri M. Falkner D. H. Morel P. A. (2002). Regulatory Th2 response induced following adoptive transfer of dendritic cells in prediabetic NOD mice. Eur. J. Immunol. 32, 2021–2030 10.1002/1521-4141(200207)32:7<2021::AID-IMMU2021>3.0.CO;2-J 12115623

[B63] Flohe’ S. B. Bruggemann J. Lendemans S. Nikulina M. Meierhoff G. Flohe’ S. (2003). Human heat shock protein 60 induces maturation of dendritic cells versus a Th1-promoting phenotype. J. Immunol. 170, 2340–2348 12594256 10.4049/jimmunol.170.5.2340

[B64] Frisoni L. Mcphie L. Colonna L. Sriram U. Monestier M. Gallucci S. (2005). Nuclear autoantigen translocation and autoantibody opsonization lead to increased dendritic cell phagocytosis and presentation of nuclear antigens: a novel pathogenic pathway for autoimmunity? J. Immunol. 175, 2692–2701 16081846 10.4049/jimmunol.175.4.2692

[B65] Furnrohr B. G. Wach S. Kelly J. A. Haslbeck M. Weber C. K. Stach C. M. (2010). Polymorphisms in the Hsp70 gene locus are genetically associated with systemic lupus erythematosus. Ann. Rheum. Dis. 69, 1983–1989 10.1136/ard.2009.122630 20498198 PMC3002760

[B66] Gall A. Treuting P. Elkon K. B. Loo Y. M. Gale M. Jr. Barber G. N. (2012). Autoimmunity initiates in nonhematopoietic cells and progresses via lymphocytes in an interferon-dependent autoimmune disease. Immunity 36, 120–131 10.1016/j.immuni.2011.11.018 22284419 PMC3269499

[B67] Gallucci S. Lolkema M. Matzinger P. (1999). Natural adjuvants: endogenous activators of dendritic cells. Nat. Med. 5, 1249–1255 10.1038/15200 10545990

[B68] Gallucci S. Matzinger P. (2001). Danger signals: SOS to the immune system. Curr. Opin. Immunol. 13, 114–119 10.1016/S0952-7915(00)00191-6 11154927

[B69] Galluzzi L. Kepp O. Kroemer G. (2012). Mitochondria: master regulators of danger signalling. Nat. Rev. Mol. Cell Biol. 13, 780–788 10.1038/nrm3479 23175281

[B70] Garber S. S. Cahalan M. D. (1997). Volume-regulated anion channels and the control of a simple cell behavior. Cell. Physiol. Biochem. 7, 229–241 10.1159/000154877

[B71] Garcia-Romo G. S. Caielli S. Vega B. Connolly J. Allantaz F. Xu Z. (2011). Netting neutrophils are major inducers of type I IFN production in pediatric systemic lupus erythematosus. Sci. Transl. Med. 3, 73ra20 10.1126/scitranslmed.3001201 21389264 PMC3143837

[B72] García-Vallejo J. Ambrosini M. Overbeek A. Van Riel W. Bloem K. Unger W. (2013). Multivalent glycopeptide dendrimers for the targeted delivery of antigens to dendritic cells. Mol. Immunol. 53, 387–397 10.1016/j.molimm.2012.09.012 23103377

[B73] Gardella S. Andrei C. Ferrera D. Lotti L. V. Torrisi M. R. Bianchi M. E. (2002). The nuclear protein HMGB1 is secreted by monocytes via a non-classical, vesicle-mediated secretory pathway. EMBO Rep. 3, 995–1001 10.1093/embo-reports/kvf198 12231511 PMC1307617

[B74] Gergely P. Jr. Grossman C. Niland B. Puskas F. Neupane H. Allam F. (2002). Mitochondrial hyperpolarization and ATP depletion in patients with systemic lupus erythematosus. Arthritis Rheum. 46, 175–190 10.1002/1529-0131(200201)46:1<175::AID-ART10015>3.0.CO;2-H 11817589 PMC4020417

[B75] Germain R. N. (2012). Maintaining system homeostasis: the third law of Newtonian immunology. Nat. Immunol. 13, 902–906 10.1038/ni.2404 22990887 PMC3518435

[B76] Gerner M. Y. Kastenmuller W. Ifrim I. Kabat J. Germain R. N. (2012). Histo-cytometry: a method for highly multiplex quantitative tissue imaging analysis applied to dendritic cell subset microanatomy in lymph nodes. Immunity 37, 364–376 10.1016/j.immuni.2012.07.011 22863836 PMC3514885

[B77] Ghiringhelli F. Apetoh L. Tesniere A. Aymeric L. Ma Y. Ortiz C. (2009). Activation of the NLRP3 inflammasome in dendritic cells induces IL-1Œ ≤,Äí dependent adaptive immunity against tumors. Nat. Med. 15, 1170–1178 10.1038/nm.2028 19767732

[B78] Ginhoux F. Ng L. G. Merad M. (2012). Understanding the murine cutaneous dendritic cell network to improve intradermal vaccination strategies. Curr. Top. Microbiol. Immunol. 351, 1–24 10.1007/82_2010_115 21058006

[B79] Gloire G. Legrand-Poels S. Piette J. (2006). NF-kappaB activation by reactive oxygen species: fifteen years later. Biochem. Pharmacol. 72, 1493–1505 10.1016/j.bcp.2006.04.011 16723122

[B80] Goh F. G. Midwood K. S. (2011). Intrinsic danger: activation of Toll-like receptors in rheumatoid arthritis. Rheumatology (Oxford) 51, 7–23 10.1093/rheumatology/ker257 21984766

[B81] Goldstein R. S. Gallowitsch-Puerta M. Yang L. Rosas-Ballina M. Huston J. M. Czura C. J. (2006). Elevated high-mobility group box 1 levels in patients with cerebral and myocardial ischemia. Shock 25, 571–574 10.1097/01.shk.0000209540.99176.72 16721263

[B82] Gottfried E. Kunz-Schughart L. A. Ebner S. Mueller-Klieser W. Hoves S. Andreesen R. (2006). Tumor-derived lactic acid modulates dendritic cell activation and antigen expression. Blood 107, 2013–2021 10.1182/blood-2005-05-1795 16278308

[B83] Gris D. Ye Z. Iocca H. A. Wen H. Craven R. R. Gris P. (2010). NLRP3 plays a critical role in the development of experimental autoimmune encephalomyelitis by mediating Th1 and Th17 responses. J. Immunol. 185, 974–981 10.4049/jimmunol.0904145 20574004 PMC3593010

[B84] Grivennikov S. I. (2013). Inflammation and colorectal cancer: colitis-associated neoplasia. Semin. Immunopathol. 35, 229–244 10.1007/s00281-012-0352-6 23161445 PMC3568220

[B85] Gunther C. Meurer M. Stein A. Viehweg A. Lee-Kirsch M. A. (2009). Familial chilblain lupus – a monogenic form of cutaneous lupus erythematosus due to a heterozygous mutation in TREX1. Dermatology (Basel) 219, 162–166 10.1159/000222430 19478477

[B86] Hammer G. E. Ma A. (2013). Molecular control of steady-state dendritic cell maturation and immune homeostasis. Annu. Rev. Immunol. 31, 743–791 10.1146/annurev-immunol-020711-074929 23330953 PMC4091962

[B87] Han J. Zhong J. Wei W. Wang Y. Huang Y. Yang P. (2008). Extracellular high-mobility group box 1 acts as an innate immune mediator to enhance autoimmune progression and diabetes onset in NOD mice. Diabetes 57, 2118–2127 10.2337/db07-1499 18477810 PMC2494682

[B88] Harley I. T. Kaufman K. M. Langefeld C. D. Harley J. B. Kelly J. A. (2009). Genetic susceptibility to SLE: new insights from fine mapping and genome-wide association studies. Nat. Rev. Genet. 10, 285–290 10.1038/nrg2571 19337289 PMC2737697

[B89] Harris H. E. Andersson U. Pisetsky D. S. (2012). HMGB1: a multifunctional alarmin driving autoimmune and inflammatory disease. Nat Rev Rheumatol 8, 195–202 10.1038/nrrheum.2011.222 22293756

[B90] Hartmann H. Eltzschig H. K. Wurz H. Hantke K. Rakin A. Yazdi A. S. (2008). Hypoxia-independent activation of HIF-1 by enterobacteriaceae and their siderophores. Gastroenterology 134, 756–767 10.1053/j.gastro.2007.12.008 18325389

[B91] Heil M. Ibarra-Laclette E. Adame-Alvarez R. M. Martinez O. Ramirez-Chavez E. Molina-Torres J. (2012). How plants sense wounds: damaged-self recognition is based on plant-derived elicitors and induces octadecanoid signaling. PLoS ONE 7:e30537 10.1371/journal.pone.0030537 22347382 PMC3276496

[B92] Hilbi H. (2009). Bacterial jailbreak sounds cellular alarm: phagosome membrane remnants trigger signaling. Cell Host Microbe 6, 102–104 10.1016/j.chom.2009.08.001 19683675

[B93] Hollander A. P. Corke K. P. Freemont A. J. Lewis C. E. (2001). Expression of hypoxia-inducible factor 1alpha by macrophages in the rheumatoid synovium: implications for targeting of therapeutic genes to the inflamed joint. Arthritis Rheum. 44, 1540–1544 10.1002/1529-0131(200107)44:7¡1540::AID-ART277.3.0.CO;2-7 11465705

[B94] Hornung V. Bauernfeind F. Halle A. Samstad E. O. Kono H. Rock K. L. (2008). Silica crystals and aluminum salts activate the NALP3 inflammasome through phagosomal destabilization. Nat. Immunol. 9, 847–856 10.1038/ni.1631 18604214 PMC2834784

[B95] Hur J. W. Sung Y. K. Shin H. D. Park B. L. Cheong H. S. Bae S. C. (2008). TREX1 polymorphisms associated with autoantibodies in patients with systemic lupus erythematosus. Rheumatol. Int. 28, 783–789 10.1007/s00296-007-0509-0 18092167

[B96] Hwang I. I. L. Watson I. R. Der S. D. Ohh M. (2006). Loss of VHL confers hypoxia-inducible factor (HIF)-dependent resistance to vesicular stomatitis virus: role of HIF in antiviral response. J. Virol. 80, 10712–10723 10.1128/JVI.01014-06 16928739 PMC1641802

[B97] Ichimonji I. Tomura H. Mogi C. Sato K. Aoki H. Hisada T. (2010). Extracellular acidification stimulates IL-6 production and Ca(2+) mobilization through proton-sensing OGR1 receptors in human airway smooth muscle cells. Am. J. Physiol. Lung Cell Mol. Physiol. 299, L567–L577 10.1152/ajplung.00415.2009 20656891

[B98] Iyer S. S. Pulskens W. P. Sadler J. J. Butter L. M. Teske G. J. Ulland T. K. (2009). Necrotic cells trigger a sterile inflammatory response through the Nlrp3 inflammasome. Proc. Natl. Acad. Sci. U.S.A. 106, 20388–20393 10.1073/pnas.0908698106 19918053 PMC2787135

[B99] Jancic C. C. Cabrini M. Gabelloni M. L. Rodríguez Rodrigues C. Salamone G. Trevani A. S. (2011). Low extracellular pH stimulates the production of IL-1Œ ≤ by human monocytes. Cytokine 57, 258–268 10.1016/j.cyto.2011.11.013 22154780

[B100] Janeway C. A. Jr. (1992). The immune system evolved to discriminate infectious nonself from noninfectious self. Immunol. Today 13, 11–16 10.1016/0167-5699(92)90198-G 1739426

[B101] Janeway C. J. (1989). Approaching the asymptote? Evolution and revolution in immunology. Cold Spring Harb. Symp. Quant. Biol. 54, 1–13 10.1101/SQB.1989.054.01.003 2700931

[B102] Jantsch J. Chakravortty D. Turza N. Prechtel A. T. Buchholz B. R. Gerlach R. G. (2008). Hypoxia and hypoxia-inducible factor-1 alpha modulate lipopolysaccharide-induced dendritic cell activation and function. J. Immunol. 180, 4697–4705 18354193 10.4049/jimmunol.180.7.4697

[B103] Javaherian K. Liu J. F. Wang J. C. (1978). Nonhistone proteins HMG1 and HMG2 change the DNA helical structure. Science 199, 1345–1346 10.1126/science.628842 628842

[B104] Jeong H.-J. Chung H.-S. Lee B.-R. Kim S.-J. Yoo S.-J. Hong S.-H. (2003). Expression of proinflammatory cytokines via HIF-1alpha and NF-kappaB activation on desferrioxamine-stimulated HMC-1 cells. Biochem. Biophys. Res. Commun. 306, 805–811 10.1016/S0006-291X(03)01073-8 12821113

[B105] Jiang W. Pisetsky D. S. (2008). Expression of high mobility group protein 1 in the sera of patients and mice with systemic lupus erythematosus. Ann. Rheum. Dis. 67, 727–728 10.1136/ard.2007.074484 18408114

[B106] Jouan-Lanhouet S. Arshad M. I. Piquet-Pellorce C. Martin-Chouly C. Le Moigne-Muller G. Van Herreweghe F. (2012). TRAIL induces necroptosis involving RIPK1/RIPK3-dependent PARP-1 activation. Cell Death Differ. 19, 2003–2014 10.1038/cdd.2012.90 22814620 PMC3504714

[B107] Julian M. Shao G. Bao S. Knoell D. Papenfuss T. Vangundy Z. (2012). Mitochondrial transcription factor A serves as a danger signal by augmenting plasmacytoid dendritic cell responses to DNA. J. immunol. 189, 433–443 10.4049/jimmunol.1101375 22675199 PMC3381894

[B108] Kaba N. K. Knauf P. A. (2001). Hypotonicity induces L-selectin shedding in human neutrophils. Am. J. Physiol. Cell Physiol. 281, C1403–C1407 11546679 10.1152/ajpcell.2001.281.4.C1403

[B109] Kalled S. L. Cutler A. H. Burkly L. C. (2001). Apoptosis and altered dendritic cell homeostasis in lupus nephritis are limited by anti-CD154 treatment. J. Immunol. 167, 1740–1747 11466399 10.4049/jimmunol.167.3.1740

[B110] Katzourakis A. Gifford R. J. (2010). Endogenous viral elements in animal genomes. PLoS Genet. 6:e1001191 10.1371/journal.pgen.1001191 21124940 PMC2987831

[B111] Kim J. Mooney D. J. (2011). In vivo modulation of dendritic cells by engineered materials: towards new cancer vaccines. Nano Today 6, 466–477 10.1016/j.nantod.2011.08.005 22125572 PMC3224090

[B112] Kleinewietfeld M. Manzel A. Titze J. Kvakan H. Yosef N. Linker R. (2013). Sodium chloride drives autoimmune disease by the induction of pathogenic TH17 cells. Nature 496, 518–522 10.1038/nature11868 23467095 PMC3746493

[B113] Knippertz I. Stein M. F. Dorrie J. Schaft N. Muller I. Deinzer A. (2011). Mild hyperthermia enhances human monocyte-derived dendritic cell functions and offers potential for applications in vaccination strategies. Int. J. Hyperthermia 27, 591–603 10.3109/02656736.2011.589234 21846195

[B114] Koike E. Takano H. Inoue K.-I. Yanagisawa R. Kobayashi T. (2008). Carbon black nanoparticles promote the maturation and function of mouse bone marrow-derived dendritic cells. Chemosphere 73, 371–376 10.1016/j.chemosphere.2008.05.054 18602660

[B115] Kojima H. Gu H. Nomura S. Caldwell C. C. Kobata T. Carmeliet P. (2002). Abnormal B lymphocyte development and autoimmunity in hypoxia-inducible factor 1alpha-deficient chimeric mice. Proc. Natl. Acad. Sci. U.S.A. 99, 2170–2174 10.1073/pnas.052706699 11854513 PMC122337

[B116] Kokkola R. Andersson A. Mullins G. Ostberg T. Treutiger C. J. Arnold B. (2005). RAGE is the major receptor for the proinflammatory activity of HMGB1 in rodent macrophages. Scand. J. Immunol. 61, 1–9 10.1111/j.0300-9475.2005.01534.x 15644117

[B117] Kokkola R. Li J. Sundberg E. Aveberger A. C. Palmblad K. Yang H. (2003). Successful treatment of collagen-induced arthritis in mice and rats by targeting extracellular high mobility group box chromosomal protein 1 activity. Arthritis Rheum. 48, 2052–2058 10.1002/art.11161 12847700

[B118] Konttinen Y. T. Mackiewicz Z. Povilenaite D. Sukura A. Hukkanen M. Virtanen I. (2004). Disease-associated increased HIF-1, alphavbeta3 integrin, and Flt-1 do not suffice to compensate the damage-inducing loss of blood vessels in inflammatory myopathies. Rheumatol. Int. 24, 333–339 10.1007/s00296-003-0379-z 13680145

[B119] Krysko D. V. Agostinis P. Krysko O. Garg A. D. Bachert C. Lambrecht B. N. (2011). Emerging role of damage-associated molecular patterns derived from mitochondria in inflammation. Trends Immunol. 32, 157–164 10.1016/j.it.2011.01.005 21334975

[B120] Kuhlicke J. Frick J. S. Morote-Garcia J. C. Rosenberger P. Eltzschig H. K. (2007). Hypoxia inducible factor (HIF)-1 coordinates induction of Toll-like receptors TLR2 and TLR6 during hypoxia. PLoS ONE 2:e1364 10.1371/journal.pone.0001364 18159247 PMC2147045

[B121] Kwak D. Choi S. Jeong H. Jang J.-H. Lee Y. Jeon H. (2012). Osmotic stress regulates mammalian target of rapamycin (mTOR) complex 1 via c-Jun N-terminal Kinase (JNK)-mediated Raptor protein phosphorylation. J. Biol. Chem. 287, 18398–18407 10.1074/jbc.M111.326538 22493283 PMC3365776

[B122] Lamkanfi M. Dixit V. M. (2012). Inflammasomes and their roles in health and disease. Annu. Rev. Cell Dev. Biol. 28, 137–161 10.1146/annurev-cellbio-101011-155745 22974247

[B123] Land W. G. Messmer K. (2012). The danger theory in view of the injury hypothesis: 20 years later. Front. Immunol. 3:349 10.3389/fimmu.2012.00349 23189080 PMC3505962

[B124] Lang P. Merkler D. Funkner P. Shaabani N. Meryk A. Krings C. (2010). Oxidized ATP inhibits T-cell-mediated autoimmunity. Eur. J. Immunol. 40, 2401–2408 10.1002/eji.200939838 20683833

[B125] Lardner A. (2001). The effects of extracellular pH on immune function. J. Leukoc. Biol. 69, 522–530 11310837

[B126] Leadbetter E. A. Rifkin I. R. Hohlbaum A. M. Beaudette B. C. Shlomchik M. J. Marshak-Rothstein A. (2002). Chromatin-IgG complexes activate B cells by dual engagement of IgM and Toll-like receptors. Nature 416, 603–607 10.1038/416603a 11948342

[B127] Lee-Kirsch M. A. Chowdhury D. Harvey S. Gong M. Senenko L. Engel K. (2007a). A mutation in TREX1 that impairs susceptibility to granzyme A-mediated cell death underlies familial chilblain lupus. J. Mol. Med. (Berl.) 85, 531–537 10.1007/s00109-007-0199-9 17440703

[B128] Lee-Kirsch M. A. Gong M. Chowdhury D. Senenko L. Engel K. Lee Y. A. (2007b). Mutations in the gene encoding the 3′-5′ DNA exonuclease TREX1 are associated with systemic lupus erythematosus. Nat. Genet. 39, 1065–1067 10.1038/ng2091 17660818

[B129] Li A. Qin L. Zhu D. Zhu R. Sun J. Wang S. (2010). Signalling pathways involved in the activation of dendritic cells by layered double hydroxide nanoparticles. Biomaterials 31, 748–756 10.1016/j.biomaterials.2009.09.095 19853910

[B130] Lin M. Yiu W. Wu H. Chan L. Y. Leung J. Au W. (2012). Toll-like receptor 4 promotes tubular inflammation in diabetic nephropathy. J. Am. Soc. Nephrol. 23, 86–102 10.1681/ASN.2010111210 22021706 PMC3269929

[B131] Liu B. Dai J. Zheng H. Stoilova D. Sun S. Li Z. (2003). Cell surface expression of an endoplasmic reticulum resident heat shock protein gp96 triggers MyD88-dependent systemic autoimmune diseases. Proc. Natl. Acad. Sci. U.S.A. 100, 15824–15829 10.1073/pnas.2635458100 14668429 PMC307652

[B132] Liu Y. J. (2005). IPC: professional type 1 interferon-producing cells and plasmacytoid dendritic cell precursors. Annu. Rev. Immunol. 23, 275–306 10.1146/annurev.immunol.23.021704.115633 15771572

[B133] Lotze M. T. Tracey K. J. (2005). High-mobility group box 1 protein (HMGB1): nuclear weapon in the immune arsenal. Nat. Rev. Immunol. 5, 331–342 10.1038/nri1594 15803152

[B134] Maeno E. Ishizaki Y. Kanaseki T. Hazama A. Okada Y. (2000). Normotonic cell shrinkage because of disordered volume regulation is an early prerequisite to apoptosis. Proc. Natl. Acad. Sci. U.S.A. 97, 9487–9492 10.1073/pnas.140216197 10900263 PMC16891

[B135] Maeno E. Shimizu T. Okada Y. (2006). Normotonic cell shrinkage induces apoptosis under extracellular low Cl conditions in human lymphoid and epithelial cells. Acta Physiol (Oxf.) 187, 217–222 10.1111/j.1748-1716.2006.01554.x 16734758

[B136] Mancino A. Schioppa T. Larghi P. Pasqualini F. Nebuloni M. Chen I. H. (2008). Divergent effects of hypoxia on dendritic cell functions. Blood 112, 3723–3734 10.1182/blood-2008-02-142091 18694997

[B137] Manolova V. Flace A. Bauer M. Schwarz K. Saudan P. Bachmann M. F. (2008). Nanoparticles target distinct dendritic cell populations according to their size. Eur. J. Immunol. 38, 1404–1413 10.1002/eji.200737984 18389478

[B138] Mariathasan S. Newton K. Monack D. Vucic D. French D. Lee W. (2004). Differential activation of the inflammasome by caspase-1 adaptors ASC and Ipaf. Nature 430, 213–218 10.1038/nature02664 15190255

[B139] Marriott I. Inscho E. W. Bost K. L. (1999). Extracellular uridine nucleotides initiate cytokine production by murine dendritic cells. Cell. Immunol. 195, 147–156 10.1006/cimm.1999.1531 10448014

[B140] Marshak-Rothstein A. Rifkin I. R. (2007). Immunologically active autoantigens: the role of toll-like receptors in the development of chronic inflammatory disease. Annu. Rev. Immunol. 25, 419–441 10.1146/annurev.immunol.22.012703.104514 17378763

[B141] Martinez D. Vermeulen M. Trevani A. A. Ceballos A. Sabatté J. Gamberale R. (2006). Extracellular acidosis induces neutrophil activation by a mechanism dependent on activation of phosphatidylinositol 3-kinase/Akt and ERK pathways. J. Immunol. 176, 1163–1171 16394005 10.4049/jimmunol.176.2.1163

[B142] Martinez-Zaguilan R. Seftor E. A. Seftor R. E. Chu Y. W. Gillies R. J. Hendrix M. J. (1996). Acidic pH enhances the invasive behavior of human melanoma cells. Clin. Exp. Metastasis 14, 176–186 10.1007/BF00121214 8605731

[B143] Martinon F. Pétrilli V. Mayor A. Tardivel A. Tschopp J. (2006). Gout-associated uric acid crystals activate the NALP3 inflammasome. Nature 440, 237–241 10.1038/nature04516 16407889

[B144] Matzinger P. (1994). Tolerance, danger, and the extended family. Annu. Rev. Immunol. 12, 991–1045 10.1146/annurev.immunol.12.1.991 8011301

[B145] Matzinger P. (1998). An innate sense of danger. Semin. Immunol. 10, 399–415 10.1006/smim.1998.0143 9840976

[B146] Matzinger P. (2002). The danger model: a renewed sense of self. Science 296, 301–305 10.1126/science.1071059 11951032

[B147] Medzhitov R. Janeway C. A. Jr. (1997a). Innate immunity: the virtues of a nonclonal system of recognition. Cell 91, 295–298 10.1016/S0092-8674(00)80412-2 9363937

[B148] Medzhitov R. Janeway C. A. Jr. (1997b). Innate immunity: impact on the adaptive immune response. Curr. Opin. Immunol. 9, 4–9 10.1016/S0952-7915(97)80152-5 9039775

[B149] Medzhitov R. Preston-Hurlburt P. Janeway C. A. Jr. (1997). A human homologue of the Drosophila Toll protein signals activation of adaptive immunity. Nature 388, 394–397 10.1038/41131 9237759

[B150] Melli K. Friedman R. S. Martin A. E. Finger E. B. Miao G. Szot G. L. (2009). Amplification of autoimmune response through induction of dendritic cell maturation in inflamed tissues. J. Immunol. 182, 2590–2600 10.4049/jimmunol.0803543 19234153 PMC3057894

[B151] Menges M. Rossner S. Voigtlander C. Schindler H. Kukutsch N. A. Bogdan C. (2002). Repetitive injections of dendritic cells matured with tumor necrosis factor alpha induce antigen-specific protection of mice from autoimmunity. J. Exp. Med. 195, 15–21 10.1084/jem.20011341 11781361 PMC2196016

[B152] Metselaar J. M. Wauben M. H. Wagenaar-Hilbers J. P. Boerman O. C. Storm G. (2003). Complete remission of experimental arthritis by joint targeting of glucocorticoids with long-circulating liposomes. Arthritis Rheum. 48, 2059–2066 10.1002/art.11140 12847701

[B153] Millar D. G. Garza K. M. Odermatt B. Elford A. R. Ono N. Li Z. (2003). Hsp70 promotes antigen-presenting cell function and converts T-cell tolerance to autoimmunity in vivo. Nat. Med. 9, 1469–1476 10.1038/nm962 14625545

[B154] Miller J. C. Brown B. D. Shay T. Gautier E. L. Jojic V. Cohain A. (2012). Deciphering the transcriptional network of the dendritic cell lineage. Nat. Immunol. 13, 888–899 10.1038/ni.2370 22797772 PMC3985403

[B155] Mills K. H. (2011). TLR-dependent T cell activation in autoimmunity. Nat. Rev. Immunol. 11, 807–822 10.1038/nri3095 22094985

[B156] Mitchell L. A. Lauer F. T. Burchiel S. W. Mcdonald J. D. (2009). Mechanisms for how inhaled multiwalled carbon nanotubes suppress systemic immune function in mice. Nat. Nanotechnol. 4, 451–456 10.1038/nnano.2009.151 19581899 PMC3641180

[B157] Moghimi S. M. (2002). Chemical camouflage of nanospheres with a poorly reactive surface: towards development of stealth and target-specific nanocarriers. Biochim. Biophys. Acta 1590, 131–139 10.1016/S0167-4889(02)00204-5 12063176

[B158] Mohamed B. M. Verma N. K. Davies A. M. Mcgowan A. Crosbie-Staunton K. Prina-Mello A. (2012). Citrullination of proteins: a common post-translational modification pathway induced by different nanoparticles in vitro and in vivo. Nanomed. 7, 1181–1195 10.2217/nnm.11.177 22625207 PMC3465773

[B159] Morita M. Stamp G. Robins P. Dulic A. Rosewell I. Hrivnak G. (2004). Gene-targeted mice lacking the Trex1 (DNase III) 3′ →5′DNA exonuclease develop inflammatory myocarditis. Mol. Cell. Biol. 24, 6719–6727 10.1128/MCB.24.15.6719-6727.2004 15254239 PMC444847

[B160] Muller T. Robaye B. Vieira R. P. Ferrari D. Grimm M. Jakob T. (2010). The purinergic receptor P2Y2 receptor mediates chemotaxis of dendritic cells and eosinophils in allergic lung inflammation. Allergy 65, 1545–1553 10.1111/j.1398-9995.2010.02426.x 20880147

[B161] Muslimani A. Chisti M. M. Wills S. Nadeau L. Zakalik D. Daw H. (2011). How we treat tumor lysis syndrome. Oncology (Williston Park, N.Y.) 25, 369–375 21618960

[B162] Mutini C. Falzoni S. Ferrari D. Chiozzi P. Morelli A. Baricordi O. R. (1999). Mouse dendritic cells express the P2×7 purinergic receptor: characterization and possible participation in antigen presentation. J. Immunol. 163, 1958–1965 10438932

[B163] Nedergaard M. Kraig R. P. Tanabe J. Pulsinelli W. A. (1991). Dynamics of interstitial and intracellular pH in evolving brain infarct. Am. J. Physiol. 260, R581–R588 2001008 10.1152/ajpregu.1991.260.3.R581 PMC3062631

[B164] Niewold T. (2011). Interferon alpha as a primary pathogenic factor in human lupus. J. Interferon Cytokine Res. 31, 887–892 10.1089/jir.2011.0071 21923413 PMC3234490

[B165] Nin J. Ferreira I. Schalkwijk C. Jorsal A. Prins M. Parving H. H. (2012a). Higher plasma high-mobility group box 1 levels are associated with incident cardiovascular disease and all-cause mortality in type 1 diabetes: a 12 year follow-up study. Diabetologia 55, 2489–2493 10.1007/s00125-012-2622-1 22752054 PMC3411294

[B166] Nin J. Ferreira I. Schalkwijk C. Prins M. Chaturvedi N. Fuller J. (2012b). Serum high-mobility group box-1 levels are positively associated with micro- and macroalbuminuria but not with cardiovascular disease in type 1 diabetes: the EURODIAB Prospective Complications Study. Eur. J. Endocrinol. 166, 325–332 10.1530/EJE-11-0662 22127490

[B167] Nizet V. Johnson R. S. (2009). Interdependence of hypoxic and innate immune responses. Nat. Rev. Immunol. 9, 609–617 10.1038/nri2607 19704417 PMC4343208

[B168] Oates J. C. Gilkeson G. S. (2006). The biology of nitric oxide and other reactive intermediates in systemic lupus erythematosus. Clin. Immunol. 121, 243–250 10.1016/j.clim.2006.06.001 16861040 PMC2765327

[B169] Odegaard J. Chawla A. (2013). The immune system as a sensor of the metabolic state. Immunity 38, 644–654 10.1016/j.immuni.2013.04.001 23601683 PMC3663597

[B170] Ohnmacht C. Pullner A. King S. B. Drexler I. Meier S. Brocker T. (2009). Constitutive ablation of dendritic cells breaks self-tolerance of CD4 T cells and results in spontaneous fatal autoimmunity. J. Exp. Med. 206, 549–559 10.1084/jem.20082394 19237601 PMC2699126

[B171] Park J. S. Svetkauskaite D. He Q. Kim J.-Y. Strassheim D. Ishizaka A. (2004). Involvement of toll-like receptors 2 and 4 in cellular activation by high mobility group box 1 protein. J. Biol. Chem. 279, 7370–7377 10.1074/jbc.M306793200 14660645

[B172] Pearce E. Pearce E. (2013). Metabolic pathways in immune cell activation and quiescence. Immunity 38, 633–643 10.1016/j.immuni.2013.04.005 23601682 PMC3654249

[B173] Perl A. Gergely P. Jr Nagy G. Koncz A. Banki K. (2004). Mitochondrial hyperpolarization: a checkpoint of T-cell life, death and autoimmunity. Trends Immunol. 25, 360–367 10.1016/j.it.2004.05.001 15207503 PMC4034110

[B174] Perl A. Hanczko R. Doherty E. (2012). Assessment of mitochondrial dysfunction in lymphocytes of patients with systemic lupus erythematosus. Methods Mol. Biol. 900, 61–89 10.1007/978-1-60761-720-4_4 22933065

[B175] Peyssonnaux C. Datta V. Cramer T. Doedens A. Theodorakis E. A. Gallo R. L. (2005). HIF-1alpha expression regulates the bactericidal capacity of phagocytes. J. Clin. Invest. 115, 1806–1815 10.1172/JCI23865 16007254 PMC1159132

[B176] Pichlmair A. Reis e Sousa C. (2007). Innate recognition of viruses. Immunity 27, 370–383 10.1016/j.immuni.2007.08.012 17892846

[B177] Pisitkun P. Deane J. A. Difilippantonio M. J. Tarasenko T. Satterthwaite A. B. Bolland S. (2006). Autoreactive B cell responses to RNA-related antigens due to TLR7 gene duplication. Science 312, 1669–1672 10.1126/science.1124978 16709748

[B178] Pociot F. Ronningen K. S. Nerup J. (1993). Polymorphic analysis of the human MHC-linked heat shock protein 70 (HSP70-2) and HSP70-Hom genes in insulin-dependent diabetes mellitus (IDDM). Scand. J. Immunol. 38, 491–495 10.1111/j.1365-3083.1993.tb02593.x 7901896

[B179] Poltorak A. He X. Smirnova I. Liu M. Y. Van Huffel C. Du X. (1998). Defective LPS signaling in C3H/HeJ and C57BL/10ScCr mice: mutations in Tlr4 gene. Science 282, 2085–2088 10.1126/science.282.5396.2085 9851930

[B180] Ramachandran S. Bell R. B. (1995). Heat shock protein 70 gene polymorphisms and multiple sclerosis. Tissue Antigens 46, 140–141 10.1111/j.1399-0039.1995.tb02492.x 7482509

[B181] Ratanachaiyavong S. Demaine A. G. Campbell R. D. Mcgregor A. M. (1991). Heat shock protein 70 (HSP70) and complement C4 genotypes in patients with hyperthyroid Graves’ disease. Clin. Exp. Immunol. 84, 48–52 1673096 PMC1535356

[B182] Rath E. Haller D. (2012). Mitochondria at the interface between danger signaling and metabolism: role of unfolded protein responses in chronic inflammation. Inflamm. Bowel Dis. 18, 1364–1377 10.1002/ibd.21944 22183876

[B183] Reis e Sousa C. (2006). Dendritic cells in a mature age. Nat. Rev. Immunol. 6, 476–483 10.1038/nri1845 16691244

[B184] Rescigno M. (2010). Intestinal dendritic cells. Adv. Immunol. 107, 109–138 10.1016/B978-0-12-381300-8.00004-6 21034972

[B185] Ridge J. P. Di Rosa F. Matzinger P. (1998). A conditioned dendritic cell can be a temporal bridge between a CD4+ T-helper and a T-killer cell. Nature 393, 474–478 10.1038/30989 9624003

[B186] Riteau N. Baron L. Villeret B. Guillou N. Savigny F. Ryffel B. (2012). ATP release and purinergic signaling: a common pathway for particle-mediated inflammasome activation. Cell Death Dis. 3, e403 10.1038/cddis.2012.144 23059822 PMC3481132

[B187] Robinson A. P. Caldis M. W. Harp C. T. Goings G. E. Miller S. D. (2013). High-mobility group box 1 protein (HMGB1) neutralization ameliorates experimental autoimmune encephalomyelitis. J. Autoimmun. 43, 32–43 10.1016/j.jaut.2013.02.005 23514872 PMC3672339

[B188] Robson S. Sévigny J. Zimmermann H. (2006). The E-NTPDase family of ectonucleotidases: structure function relationships and pathophysiological significance. Purinergic Signal. 2, 409–430 10.1007/s11302-006-9003-5 18404480 PMC2254478

[B189] Rock K. L. Kataoka H. Lai J. J. (2013). Uric acid as a danger signal in gout and its comorbidities. Nat Rev Rheumatol 9, 13–23 10.1038/nrrheum.2012.143 22945591 PMC3648987

[B190] Rock K. L. Kono H. (2008). The inflammatory response to cell death. Annu. Rev. Pathol. 3, 99–126 10.1146/annurev.pathmechdis.3.121806.151456 18039143 PMC3094097

[B191] Rock K. L. Latz E. Ontiveros F. Kono H. (2010). The sterile inflammatory response. Annu. Rev. Immunol. 28, 321–342 10.1146/annurev-immunol-030409-101311 20307211 PMC4315152

[B192] Rofstad E. K. Mathiesen B. Kindem K. Galappathi K. (2006). Acidic extracellular pH promotes experimental metastasis of human melanoma cells in athymic nude mice. Cancer Res. 66, 6699–6707 10.1158/0008-5472.CAN-06-0983 16818644

[B193] Rosmalen J. G. Homo-Delarche F. Durant S. Kap M. Leenen P. J. Drexhage H. A. (2000). Islet abnormalities associated with an early influx of dendritic cells and macrophages in NOD and NODscid mice. Lab. Invest. 80, 769–777 10.1038/labinvest.3780080 10830787

[B194] Sallusto F. Cella M. Danieli C. Lanzavecchia A. (1995). Dendritic cells use macropinocytosis and the mannose receptor to concentrate macromolecules in the major histocompatibility complex class II compartment: downregulation by cytokines and bacterial products. J. Exp. Med. 182, 389–400 10.1084/jem.182.2.389 7629501 PMC2192110

[B195] Salvador-Morales C. Flahaut E. Sim E. Sloan J. Green M. L. H. Sim R. B. (2006). Complement activation and protein adsorption by carbon nanotubes. Mol. Immunol. 43, 193–201 10.1016/j.molimm.2005.02.006 16199256

[B196] Santiago-Raber M. L. Baudino L. Izui S. (2009). Emerging roles of TLR7 and TLR9 in murine SLE. J. Autoimmun. 33, 231–238 10.1016/j.jaut.2009.10.001 19846276

[B197] Sauter B. Albert M. L. Francisco L. Larsson M. Somersan S. Bhardwaj N. (2000). Consequences of cell death: exposure to necrotic tumor cells, but not primary tissue cells or apoptotic cells, induces the maturation of immunostimulatory dendritic cells [see comments]. J. Exp. Med. 191, 423–434 10.1084/jem.191.3.423 10662788 PMC2195816

[B198] Scaffidi P. Misteli T. Bianchi M. E. (2002). Release of chromatin protein HMGB1 by necrotic cells triggers inflammation. Nature 418, 191–195 10.1038/nature00858 12110890

[B199] Schiraldi M. Monestier M. (2009). How can a chemical element elicit complex immunopathology? Lessons from mercury-induced autoimmunity. Trends Immunol. 30, 502–509 10.1016/j.it.2009.07.005 19709928

[B200] Schiraldi M. Raucci A. Munoz L. M. Livoti E. Celona B. Venereau E. (2012). HMGB1 promotes recruitment of inflammatory cells to damaged tissues by forming a complex with CXCL12 and signaling via CXCR4. J. Exp. Med. 209, 551–563 10.1084/jem.20111739 22370717 PMC3302219

[B201] Schorn C. Strysio M. Janko C. Munoz L. E. Schett G. Herrmann M. (2010). The uptake by blood-borne phagocytes of monosodium urate is dependent on heat-labile serum factor(s) and divalent cations. Autoimmunity 43, 236–238 10.3109/08916930903510948 20187703

[B202] Semenza G. L. (2009). Regulation of oxygen homeostasis by hypoxia-inducible factor 1. Physiology (Bethesda) 24, 97–106 10.1152/physiol.00045.2008 19364912

[B203] Seong S.-Y. Matzinger P. (2004). Opinion: hydrophobicity: an ancient damage-associated molecular pattern that initiates innate immune responses. Nat. Rev. Immunol. 4, 469–478 10.1038/nri1372 15173835

[B204] Sethi V. Rubinstein I. Kuzmis A. Kastrissios H. Artwohl J. Onyuksel H. (2013). Novel, biocompatible, and disease modifying VIP nanomedicine for rheumatoid arthritis. Mol. Pharm. 10, 728–738 10.1021/mp300539f 23211088 PMC3563715

[B205] Seuwen K. Ludwig M.-G. Wolf R. M. (2006). Receptors for protons or lipid messengers or both? J. Recept. Signal Transduct. Res. 26, 599–610 10.1080/10799890600932220 17118800

[B206] Shapiro L. Dinarello C. (1995). Osmotic regulation of cytokine synthesis in vitro. Proc. Natl. Acad. Sci. U.S.A. 92, 12230–12234 10.1073/pnas.92.26.12230 8618875 PMC40330

[B207] Sharp A. J. Polak P. E. Simonini V. Lin S. X. Richardson J. C. Bongarzone E. R. (2008). P2×7 deficiency suppresses development of experimental autoimmune encephalomyelitis. J. Neuroinflammation 5:33 10.1186/1742-2094-5-33 18691411 PMC2518548

[B208] Shaw P. J. Lukens J. R. Burns S. Chi H. Mcgargill M. A. Kanneganti T. D. (2010). Cutting edge: critical role for PYCARD/ASC in the development of experimental autoimmune encephalomyelitis. J. Immunol. 184, 4610–4614 10.4049/jimmunol.1000217 20368281 PMC3001131

[B209] Shi Y. Evans J. E. Rock K. L. (2003). Molecular identification of a danger signal that alerts the immune system to dying cells. Nature 425, 516–521 10.1038/nature01991 14520412

[B210] Shi Y. Zheng W. Rock K. L. (2000). Cell injury releases endogenous adjuvants that stimulate cytotoxic T cell responses. Proc. Natl. Acad. Sci. U.S.A. 97, 14590–14595 10.1073/pnas.260497597 11106387 PMC18963

[B211] Shime H. Yabu M. Akazawa T. Kodama K. Matsumoto M. Seya T. (2008). Tumor-secreted lactic acid promotes IL-23/IL-17 proinflammatory pathway. J. Immunol. 180, 7175–7183 18490716 10.4049/jimmunol.180.11.7175

[B212] Shirali A. C. Goldstein D. R. (2008). Activation of the innate immune system by the endogenous ligand hyaluronan. Curr. Opin. Organ Transplant 13, 20–25 10.1097/MOT.0b013e3282f3df04 18660702

[B213] Sica A. Melillo G. Varesio L. (2011). Hypoxia: a double-edged sword of immunity. J. Mol. Med. 89, 657–665 10.1007/s00109-011-0724-8 21336851

[B214] Simmen H. P. Blaser J. (1993). Analysis of pH and pO2 in abscesses, peritoneal fluid, and drainage fluid in the presence or absence of bacterial infection during and after abdominal surgery. Am. J. Surg. 166, 24–27 10.1016/S0002-9610(05)80576-8 8328625

[B215] Sims G. P. Rowe D. C. Rietdijk S. T. Herbst R. Coyle A. J. (2010). HMGB1 and RAGE in inflammation and cancer. Annu. Rev. Immunol. 28, 367–388 10.1146/annurev.immunol.021908.132603 20192808

[B216] Spirig R. Djafarzadeh S. Regueira T. Shaw S. G. Von Garnier C. Takala J. (2010). Effects of TLR agonists on the hypoxia-regulated transcription factor HIF-1alpha and dendritic cell maturation under normoxic conditions. PLoS ONE 5:e10983 10.1371/journal.pone.0010983 20539755 PMC2881864

[B217] Sriram U. Varghese L. Bennett H. L. Jog N. R. Shivers D. K. Ning Y. (2012). Myeloid dendritic cells from B6.NZM Sle1/Sle2/Sle3 lupus-prone mice express an IFN signature that precedes disease onset. J. Immunol. 189, 80–91 10.4049/jimmunol.1101686 22661089 PMC3381850

[B218] Srivastava P. (2002). Roles of heat-shock proteins in innate and adaptive immunity. Nat. Rev. Immunol. 2, 185–194 10.1038/nri749 11913069

[B219] Steinman R. M. Hawiger D. Nussenzweig M. C. (2003). Tolerogenic dendritic cells. Annu. Rev. Immunol. 21, 685–711 10.1146/annurev.immunol.21.120601.141040 12615891

[B220] Stern S. T. McNeil S. E. (2008). Nanotechnology safety concerns revisited. Toxicol. Sci. 101, 4–21 10.1093/toxsci/kfm169 17602205

[B221] Stetson D. B. Ko J. S. Heidmann T. Medzhitov R. (2008). Trex1 prevents cell-intrinsic initiation of autoimmunity. Cell 134, 587–598 10.1016/j.cell.2008.06.032 18724932 PMC2626626

[B222] Stocki P. Dickinson A. M. (2012). The immunosuppressive activity of heat shock protein 70. Autoimmune Dis 2012, 617213 10.1155/2012/617213 23326648 PMC3533589

[B223] Stoecklein V. M. Osuka A. Lederer J. A. (2012). Trauma equals danger – damage control by the immune system. J. Leukoc. Biol. 92, 539–551 10.1189/jlb.0212072 22654121 PMC3427603

[B224] Stoye J. P. (2012). Studies of endogenous retroviruses reveal a continuing evolutionary saga. Nat. Rev. Microbiol. 10, 395–406 10.1038/nrmicro2783 22565131

[B225] Stranger B. E. De Jager P. L. (2012). Coordinating GWAS results with gene expression in a systems immunologic paradigm in autoimmunity. Curr. Opin. Immunol. 24, 544–551 10.1016/j.coi.2012.09.002 23040211 PMC3489007

[B226] Tang D. Billiar T. Lotze M. (2012). A Janus tale of two active high mobility group box 1 (HMGB1) redox states. Mol. Med. 18, 1360–1362 10.2119/molmed.2012.00314 23073660 PMC3533642

[B227] Taniguchi T. Takaoka A. (2002). The interferon-alpha/beta system in antiviral responses: a multimodal machinery of gene regulation by the IRF family of transcription factors. Curr. Opin. Immunol. 14, 111–116 10.1016/S0952-7915(01)00305-3 11790540

[B228] Termeer C. Benedix F. Sleeman J. Fieber C. Voith U. Ahrens T. (2002). Oligosaccharides of Hyaluronan activate dendritic cells via toll-like receptor 4. J. Exp. Med. 195, 99–111 10.1084/jem.20001858 11781369 PMC2196009

[B229] Theofilopoulos A. N. Baccala R. Beutler B. Kono D. H. (2005). Type I interferons (alpha/beta) in immunity and autoimmunity. Annu. Rev. Immunol. 23, 307–336 10.1146/annurev.immunol.23.021704.115843 15771573

[B230] Thompson M. R. Kaminski J. J. Kurt-Jones E. A. Fitzgerald K. A. (2011). Pattern recognition receptors and the innate immune response to viral infection. Viruses 3, 920–940 10.3390/v3060920 21994762 PMC3186011

[B231] Tomura H. Mogi C. Sato K. Okajima F. (2005). Proton-sensing and lysolipid-sensitive G-protein-coupled receptors: a novel type of multi-functional receptors. Cell. Signal. 17, 1466–1476 10.1016/j.cellsig.2005.06.002 16014326

[B232] Tong J. Wu W.-N. Kong X. Wu P.-F. Tian L. Du W. (2011). Acid-sensing ion channels contribute to the effect of acidosis on the function of dendritic cells. J. Immunol. 186, 3686–3692 10.4049/jimmunol.1001346 21321108

[B233] Toscano M. G. Delgado M. Kong W. Martin F. Skarica M. Ganea D. (2010). Dendritic cells transduced with lentiviral vectors expressing VIP differentiate into VIP-secreting tolerogenic-like DCs. Mol. Ther. 18, 1035–1045 10.1038/mt.2009.293 20068554 PMC2890107

[B234] Treuhaft P. S. McCarty D. J. (1971). Synovial fluid pH, lactate, oxygen and carbon dioxide partial pressure in various joint diseases. Arthritis Rheumat. 14, 475–484 5564921 10.1002/art.1780140407

[B235] Trevani A. S. Andonegui G. Giordano M. López D. H. Gamberale R. Minucci F. (1999). Extracellular acidification induces human neutrophil activation. J. Immunol. 162, 4849–4857 10202029

[B236] Ulbrich W. Lamprecht A. (2010). Targeted drug-delivery approaches by nanoparticulate carriers in the therapy of inflammatory diseases. J. R. Soc. Interface 7(Suppl. 1), S55–66 10.1098/rsif.2009.0285.focus 19940000 PMC2843985

[B237] Upton J. W. Kaiser W. J. Mocarski E. S. (2010). Virus inhibition of RIP3-dependent necrosis. Cell Host Microbe 7, 302–313 10.1016/j.chom.2010.03.006 20413098 PMC4279434

[B238] Urbonaviciute V. Furnrohr B. G. Meister S. Munoz L. Heyder P. De Marchis F. (2008). Induction of inflammatory and immune responses by HMGB1-nucleosome complexes: implications for the pathogenesis of SLE. J. Exp. Med. 205, 3007–3018 10.1084/jem.20081165 19064698 PMC2605236

[B239] Urbonaviciute V. Voll R. E. (2011). High-mobility group box 1 represents a potential marker of disease activity and novel therapeutic target in systemic lupus erythematosus. J. Intern. Med. 270, 309–318 10.1111/j.1365-2796.2011.02432.x 21793951

[B240] Vabulas R. M. Wagner H. Schild H. (2002). Heat shock proteins as ligands of toll-like receptors. Curr. Top. Microbiol. Immunol. 270, 169–184 10.1007/978-3-642-59430-4_11 12467251

[B241] Valko M. Leibfritz D. Moncol J. Cronin M. Mazur M. Telser J. (2007). Free radicals and antioxidants in normal physiological functions and human disease. Int. J. Biochem. Cell Biol. 39, 44–84 10.1016/j.biocel.2006.07.001 16978905

[B242] Vandenabeele P. Galluzzi L. Vanden Berghe T. Kroemer G. (2010). Molecular mechanisms of necroptosis: an ordered cellular explosion. Nat. Rev. Mol. Cell Biol. 11, 700–714 10.1038/nrm2970 20823910

[B243] Vermeulen M. N. Giordano M. Trevani A. S. Sedlik C. Gamberale R. Fernández-Calotti P. (2004). Acidosis improves uptake of antigens and MHC class I-restricted presentation by dendritic cells. J. Immunol. 172, 3196–3204 14978127 10.4049/jimmunol.172.5.3196

[B244] Walmsley S. R. Print C. Farahi N. Peyssonnaux C. Johnson R. S. Cramer T. (2005). Hypoxia-induced neutrophil survival is mediated by HIF-1alpha-dependent NF-kappaB activity. J. Exp. Med. 201, 105–115 10.1084/jem.20040624 15630139 PMC2212759

[B245] Wang H. Bloom O. Zhang M. Vishnubhakat J. M. Ombrellino M. Che J. (1999). HMG-1 as a late mediator of endotoxin lethality in mice. Science 285, 248–251 10.1126/science.285.5425.248 10398600

[B246] Wang Q. Liu C. Zhu F. Liu F. Zhang P. Guo C. (2010). Reoxygenation of hypoxia-differentiated dendritic cells induces Th1 and Th17 cell differentiation. Mol. Immunol. 47, 922–931 10.1016/j.molimm.2009.09.038 19910049 PMC2815172

[B247] Witte M. E. Geurts J. J. G. De Vries H. E. Van Der Valk P. Van Horssen J. (2010). Mitochondrial dysfunction: a potential link between neuroinflammation and neurodegeneration? Mitochondrion 10, 411–418 10.1016/j.mito.2010.05.014 20573557

[B248] Wu C. Yosef N. Thalhamer T. Zhu C. Xiao S. Kishi Y. (2013). Induction of pathogenic TH17 cells by inducible salt-sensing kinase SGK1. Nature 496, 513–517 10.1038/nature11984 23467085 PMC3637879

[B249] Wu T. Xie C. Han J. Ye Y. Weiel J. Li Q. (2012). Metabolic disturbances associated with systemic lupus erythematosus. PLoS ONE 7:e37210 10.1371/journal.pone.0037210 22723834 PMC3378560

[B250] Yang D. De La Rosa G. Tewary P. Oppenheim J. J. (2009). Alarmins link neutrophils and dendritic cells. Trends Immunol. 30, 531–537 10.1016/j.it.2009.07.004 19699678 PMC2767430

[B251] Yang H. Antoine D. J. Andersson U. Tracey K. J. (2013). The many faces of HMGB1: molecular structure-functional activity in inflammation, apoptosis, and chemotaxis. J. Leukoc. Biol. 93, 865–873 10.1189/jlb.1212662 23446148 PMC4051189

[B252] Yang H. Wang H. Czura C. J. Tracey K. J. (2005). The cytokine activity of HMGB1. J. Leukoc. Biol. 78, 1–8 10.1189/jlb.1104648 15734795

[B253] Yang Y. G. Lindahl T. Barnes D. E. (2007). Trex1 exonuclease degrades ssDNA to prevent chronic checkpoint activation and autoimmune disease. Cell 131, 873–886 10.1016/j.cell.2007.10.017 18045533

[B254] Yao D. Brownlee M. (2010). Hyperglycemia-induced reactive oxygen species increase expression of the receptor for advanced glycation end products (RAGE) and RAGE ligands. Diabetes 59, 249–255 10.2337/db09-0801 19833897 PMC2797929

[B255] Yeste A. Nadeau M. Burns E. J. Weiner H. L. Quintana F. J. (2012). Nanoparticle-mediated codelivery of myelin antigen and a tolerogenic small molecule suppresses experimental autoimmune encephalomyelitis. Proc. Natl. Acad. Sci. U.S.A. 109, 11270–11275 10.1073/pnas.1120611109 22745170 PMC3396465

[B256] Zeiser R. Penack O. Holler E. Idzko M. (2011). Danger signals activating innate immunity in graft-versus-host disease. J. Mol. Med. 89, 833–845 10.1007/s00109-011-0767-x 21573893

[B257] Zetterstrom C. K. Jiang W. Wahamaa H. Ostberg T. Aveberger A. C. Schierbeck H. (2008). Pivotal advance: inhibition of HMGB1 nuclear translocation as a mechanism for the anti-rheumatic effects of gold sodium thiomalate. J. Leukoc. Biol. 83, 31–38 10.1189/jlb.0507323 17913975

[B258] Zhang Q. Raoof M. Chen Y. Sumi Y. Sursal T. Junger W. (2010). Circulating mitochondrial DAMPs cause inflammatory responses to injury. Nature 464, 104–107 10.1038/nature08780 20203610 PMC2843437

[B259] Zhang S. Zhong J. Yang P. Gong F. Wang C. Y. (2009). HMGB1, an innate alarmin, in the pathogenesis of type 1 diabetes. Int. J. Clin. Exp. Pathol. 3, 24–38 19918326 PMC2776260

[B260] Zitvogel L. Kroemer G. (2010). The multifaceted granulysin. Blood 116, 3379–3380 10.1182/blood-2010-08-299214 21051561

[B261] Zolnik B. S. González-Fernández Å Sadrieh N. Dobrovolskaia M. A. (2010). Minireview: nanoparticles and the immune system. Endocrinology 151, 458–465 10.1210/en.2009-1082 20016026 PMC2817614

